# The impact of vincristine on testicular development and function in childhood cancer

**DOI:** 10.1093/humupd/dmac039

**Published:** 2022-12-10

**Authors:** Ioanna Clark, Mark F H Brougham, Norah Spears, Rod T Mitchell

**Affiliations:** MRC Centre for Reproductive Health, The Queen’s Medical Research Institute, The University of Edinburgh, Edinburgh, UK; Department of Paediatric Oncology, Royal Hospital for Children and Young People, Edinburgh, UK; Edinburgh Medical School: Biomedical Sciences, Hugh Robson Building, Edinburgh, UK; MRC Centre for Reproductive Health, The Queen’s Medical Research Institute, The University of Edinburgh, Edinburgh, UK; Department of Paediatric Endocrinology, Royal Hospital for Children and Young People, Edinburgh, UK

**Keywords:** prepuberty, childhood cancer, cancer treatment, chemotherapy, azoospermia, spermatogenic failure, testis, infertility, vincristine

## Abstract

**BACKGROUND:**

Increasing childhood cancer survival rates in recent decades have led to an increased focus on fertility as a long-term complication of cancer treatment. Male childhood cancer survivors often face compromised testicular function as a late effect of chemotherapy exposure, with no well-established options to prevent such damage and subsequent infertility. Despite vincristine being considered to be associated with low-gonadotoxic potential, in prepubertal rodents, it was recently shown to result in morphological alterations of the testis and in severely impaired fertility.

**OBJECTIVE AND RATIONALE:**

This systematic review aimed to evaluate the effects of vincristine-containing regimens on human prepubertal testis with reference to testicular function and fertility in adulthood.

**SEARCH METHODS:**

The systematic search of the literature was conducted according to PRISMA guidelines, and the study was registered with PROSPERO. PubMed and Scopus were searched for articles published in English between 01 January 1900 and 05 March 2021, with the search including ‘chemotherapy’, ‘vincristine’, ‘prepubertal’, ‘testis’, ‘spermatogenesis’ and related terms. Abstracts and full-text articles were screened and selected for, providing they met the inclusion criteria (≤12 years at treatment, exposure to vincristine-containing regimens and long-term fertility outcomes). Additional studies were identified via bibliography screening. Bias evaluation across included studies was conducted using the ROBINS-I tool, subdivided into assessment for confounding, participant selection, intervention classification, missing data, outcome measurements and selection of reported results.

**OUTCOMES:**

Our initial search identified 288 articles of which 24 (8%; n = 7134 males) met all inclusion criteria. Control groups were included for 9/24 (38%) studies and 4/24 (17%) studies provided sub-analysis of the relative gonadotoxicity of vincristine-based agents. Primary outcome measures were: fertility and parenthood; semen analysis (World Health Organization criteria); and hormonal function and testicular volume. For the studies that performed vincristine sub-analysis, none reported negative associations with vincristine for the potential of siring a pregnancy, including the largest (n = 6224; hazard ratio = 0.56) controlled study. For semen analysis, no significant difference versus healthy controls was illustrated for mitotic inhibitors (including vincristine) following sub-analysis in one study (n = 143). For hormone analysis, a single study did not find significant impacts on spermatogenesis attributed to vincristine based on levels of FSH and semen analysis, which meant that its administration was unlikely to be responsible for the diminished testicular reserve; however, most of the studies were based on low numbers of patients receiving vincristine-containing chemotherapy. Analysis of bias demonstrated that studies which included vincristine exposure sub-analysis had a lower risk of bias when compared with cohorts which did not.

**WIDER IMPLICATIONS:**

In contrast to recent findings in rodent studies, the limited number of clinical studies do not indicate gonadotoxic effects of vincristine following prepubertal exposure. However, given the relative lack of data from studies with vincristine sub-analysis, experimental studies involving vincristine exposure using human testicular tissues are warranted. Results from such studies could better inform paediatric cancer patients about their future fertility and eligibility for fertility preservation before initiation of treatment.

## Graphical Abstract

**Graphical Abstract:**
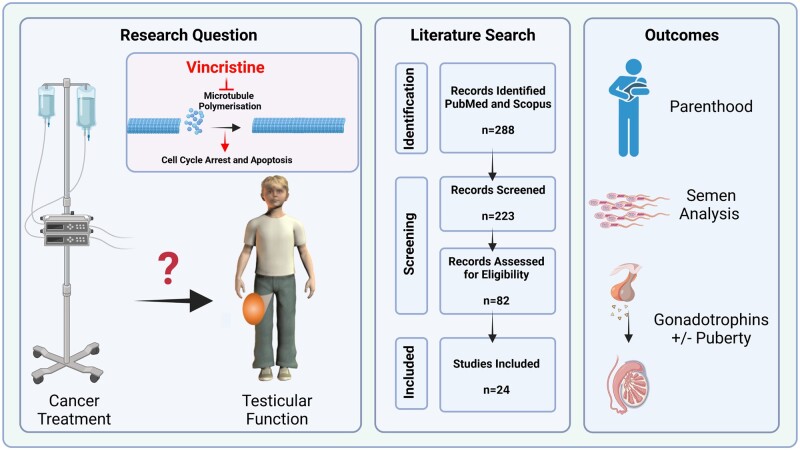
Impact of vincristine exposure in childhood on testicular development and function: summary of research question, search strategy and reproductive outcome measures.

## Introduction

Improved chemotherapy treatments that are part of anticancer regimens have significantly contributed to increasing childhood cancer survival rates in recent decades (Zebrack *et al.*, 2004). The overall current childhood cancer 5-year survival is around 80%, up from 58% in the late 1970s (Miller *et al.*, 2016). It was previously thought that the testis in prepuberty was relatively quiescent, owing to few morphological changes having been recorded and the absence of hormone production (Rey, 1999). Accordingly, the reduced testicular cell activity was believed to confer protection against insults (Rivkees and Crawford, 1988). Recent work has shown that this is not the case, with prepubertal testis undergoing important developmental changes that are required for normal adult functioning (Chow *et al.*, 2016). Long-term complications of cancer treatment in childhood survivors include infertility and other aspects of testicular dysfunction. Currently, there are no well-established options in prepubertal boys to prevent such damage and/or to preserve the potential for fertility, since unlike adult patients they cannot yet produce mature sperm for routine cryopreservation (Mitchell *et al.*, 2017). This could result in many survivors of childhood cancer being unable to father a child in the future.

For the majority of paediatric cancers, a combination of chemotherapeutic agents is required to effectively and synergistically treat malignancy (Corrie, 2011). Nonetheless, it is still poorly understood to what extent individual agents of such regimens might contribute to this damage. Vincristine is used as part of the treatment regimen for a variety of childhood cancers, including acute lymphoblastic leukaemia (ALL), brain tumours, Hodgkin’s disease and neuroblastoma and has traditionally been considered to exhibit low gonadotoxicity. As a spindle poison, its mechanism of action involves blocking the separation of chromosomes during metaphase, which could also lead to off-target side effects on healthy cells (Jordan and Wilson, 2004; Beaud *et al.*, 2017a). However, vinca alkaloids (i.e. vincristine and vinblastine) are not included in the cyclophosphamide equivalent dose (CED), which is used clinically to estimate the risks of gonadotoxicity caused by combination chemotherapy (Green *et al.*, 2014).

An unexpected effect on fertility was illustrated recently in a rodent study, which showed that exposure of prepubertal mice to vincristine at doses equivalent to those considered as having low-gonadotoxicity in humans, was associated with morphological alterations of the testicular tissue and a significant reduction in pregnancy rate (Delessard *et al.*, 2020). This suggests that the administration of vincristine during childhood cancer treatment could have long-term adverse effects on the fertility of survivors. Consequently, there is a need for a comprehensive analysis of clinical studies reporting the effects of vincristine-containing chemotherapy on gonadal development and function in children with cancer, including associations with subsequent fertility. This review aims to determine the evidence for any effects of vincristine treatment on prepubertal human testes in relation to fertility and fatherhood, sperm count and hormone production.

## Methods

A comprehensive search of published clinical studies was performed, to extract data and delineate the effects of vincristine-containing regimens on testicular development and function in childhood cancer. This review is reported according to the PRISMA guidelines for systematic reviews (Moher *et al.*, 2009). The project involves the assessment and summary of the findings of vincristine effects on fatherhood, semen analysis and perturbation of gonadotrophins. The protocol is registered on Prospero (registration number CRD42021240255) and is available from: https://www.crd.york.ac.uk/prospero/display_record.php?RecordID=240255.

### Information source

Relevant clinical studies were identified using PubMed and Scopus. Additional studies were identified through bibliographic screening of the reference lists of relevant papers and also from reviews, themselves deemed ineligible according to our criteria.

### Inclusion criteria

Inclusion criteria for study selection were:

- articles published between 01 January 1900 and 05 March 2021 and written in English;
- clinical studies involving regimens that were inclusive of vincristine chemotherapy; and
- outcomes relating to the effects of vincristine-containing regimens on fertility outcomes, i.e. fatherhood, sperm counts, reproductive hormone function (LH, FSH, testosterone) and puberty.

### Exclusion criteria

Exclusion criteria for the study selection were:

- cancer treatment exposure in which all patients were >12 years of age;
- studies that did not include human data; and
- reviews.

### Search and study selection

**Figure 1.**
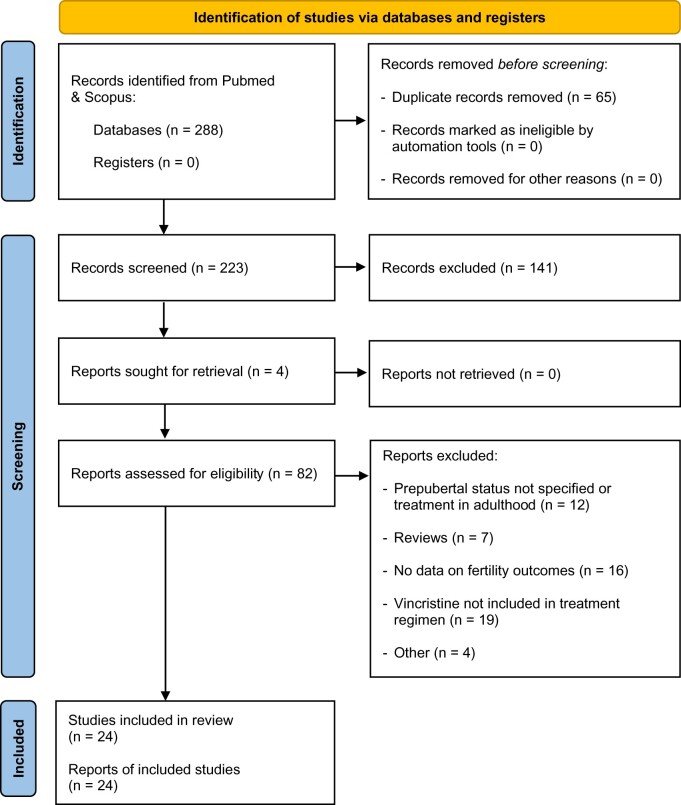
**PRISMA flow diagram for the selection of studies used to assess the effect of vincristine-containing treatment on testicular development and function in young boys with cancer.** PRISMA, preferred reporting items for systematic reviews and meta-analyses.

The search strategy and terms (Supplementary Table SI) were adapted from an earlier study (Tian En *et al.*, 2020). The number of records identified after the removal of duplicates was 223; an initial screening of titles and abstracts was required for the appropriate inclusion of studies. For this process, two assessors (R.T.M. and I.C.) applied the pre-specified eligibility criteria independently. For abstracts that were not selected by both assessors, a discussion and decision concerning the inclusion of studies for full-text screening were performed. Full texts were obtained for 82 studies and a thorough screening process discerned 58 articles that did not meet at least one aspect of the inclusion criteria (Supplementary Table SII). Twenty-four papers were included in the final analysis after a detailed assessment. A PRISMA flowchart, explaining the study selection process is shown in Fig. 1.

### Summary measures

Data extracted from the studies included outcomes, such as: partner pregnancy and fertility; semen analysis; gonadotrophin analysis; and testicular volume. One assessor (I.C.) extracted the information from the included cohorts, whilst another (R.T.M.) checked that all necessary data were successfully incorporated. Disagreements were resolved via discussion. If data were missing, study investigators were contacted for details. A comprehensive summary of all the extracted data was recorded and reported as Supplementary Material (Supplementary Table SIII).

### Quality assessment

Two assessors (R.T.M. and I.C.) evaluated the quality of individual studies based on criteria implemented via a tool for assessing internal validity in non-randomized studies (Sterne *et al.*, 2016). The risk of bias was conducted on a study level, where each study was assessed for confounding, selection of participants, intervention classification, missing data, outcome measurements and selection of reported results. Risk of bias was reported as ‘High-risk of bias identified’, ‘Low risk of bias identified’ and ‘Unclear’ (Supplementary Table SIV).

## Results

### Studies assessing testicular development and fertility post-vincristine chemotherapy

This systematic review identified 24 studies reporting testicular development and fertility post-treatment with vincristine-containing regimens (Table I). Detailed composition of the extracted data from these studies is given in Supplementary Table SIII. Of these studies, 4/24 (17%) studies provided sub-analysis of the relative gonadotoxicity of vincristine-based agents, whilst 9/24 (38%) included a control group that did not receive vincristine.

**Table I:** Summary of the 24 studies obtained in the literature search, directly relating to the use of vincristine-containing regimens, prepubertal cancer survivors and the outcome measure of reported fertility.

Study	Study design	Level of evidence*	Control group	Fertility outcome	Vincristine analysis
Kruseová *et al.* (2021)	Single-centre cohort	3	Yes	Semen analysis; gonadotrophins	Yes^c^
Utriainen *et al.* (2019)	Multicentre cohort	2	Yes^a^	Gonadotrophins; testicular volume	No
Beaud *et al.* (2019)	Multicentre cohort	2	Yes^a^	Semen analysis; gonadotrophins	Yes^c^
Green *et al.* (2014)	Multicentre cohort	3	Yes^b^	Pregnancy outcome	Yes^c^
Shiraishi and Matsuyama (2014)	Single-centre cohort^d^	3	No	Pregnancy and fertility; gonadotrophins; testicular volume	No
Hamre *et al.* (2012)	Cross-sectional study	4	No	Semen analysis; gonadotrophins; pregnancy and fertility	No
Zaletel *et al.* (2010)	Single-centre cohort	3	No	Semen analysis; gonadotrophins	No
Green *et al.* (2010)	Multicentre cohort	2	Yes^b^	Pregnancy and fertility	Yes^c^
Nurmio *et al.* (2009)	Multicentre cohort	3	No	Semen analysis; gonadotrophins; testicular volume	No
Krawczuk-Rybak *et al.* (2009)	Single-centre cohort	3	Yes^a^	Gonadotrophins; testicular volume	No
Rafsanjani *et al.* (2007)	Single-centre cohort	3	No	Semen analysis; gonadotrophins; testicular volume	No
Garolla *et al.* (2006)	Single-centre cohort	3	Yes	Semen analysis; gonadotrophins; testicular volume	No
Hobbie *et al.* (2005)	Single-centre cohort	3	No	Semen analysis; gonadotrophins; testicular volume	No
Alebouyeh *et al.* (2005)	Single-centre cohort	3	No	Semen analysis	No
Kenney *et al.* (2001)	Single-centre cohort	3	No	Semen analysis; gonadotrophins	No
Ben Arush *et al.* (2000)	Single-centre cohort	3	No	Semen analysis; gonadotrophins	No
Heikens *et al.* (1996)	Single-centre cohort	3	No	Semen analysis; gonadotrophins; testicular volume	No
Müller *et al.* (1996)	Single-centre cohort	3	Yes^a^	Semen analysis; gonadotrophins; testicular volume	No
Dhabhar *et al.* (1993)	Single-centre cohort	3	Yes^a^	Semen analysis; gonadotrophins	No
Ortin *et al.* (1990)	Single-centre cohort	3	No	Semen analysis; gonadotrophins	No
Green *et al.* (1989)	Single-centre cohort	3	No	Pregnancy and fertility	No
Jaffe *et al.* (1988)	Single-centre cohort	3	No	Semen analysis; gonadotrophins; pregnancy and fertility	No
Cramer and Andrieu (1985)	Single-centre cohort	3	No	Semen analysis	No
Ahmed *et al.* (1983)	Single-centre cohort	3	No	Gonadotrophins; testicular volume	No

*Level of evidence (as adapted from OCEBM Levels of Evidence Working Group):

1—Systematic reviews with meta-analysis of randomized clinical trials.

2—Non-randomized controlled study; prospective comparative cohort.

3—Case-control study; retrospective cohort.

4—Case series; Cross-sectional studies.

a Healthy age and sex-matched controls.

b Healthy siblings.

c Multivariate and univariate analyses.

d Patients with post-chemotherapy azoospermia.

### Quality assessment

**Figure 2.**
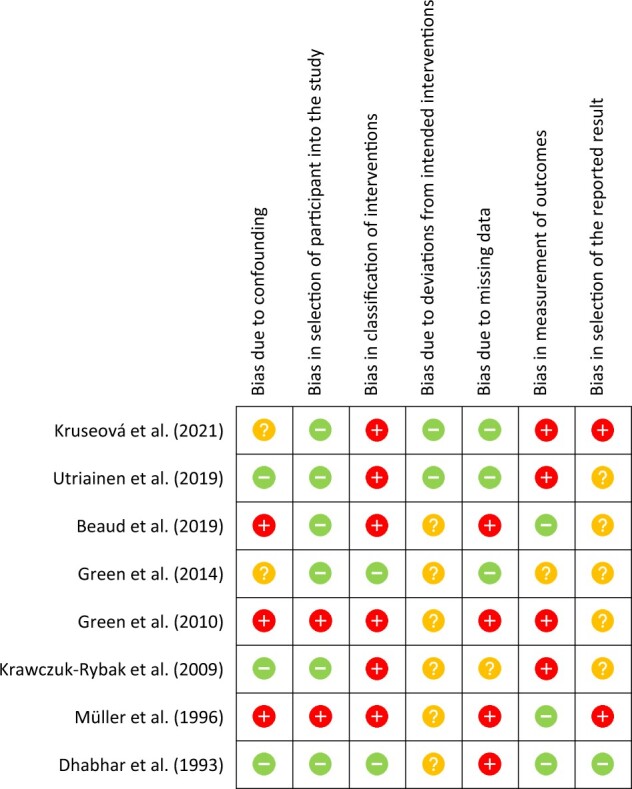
**Risk of bias summary of the eight studies included in the main data analysis.** Adapted from Eliveld *et al.* (2018). 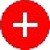 High risk of bias identified, 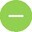 low risk of bias identified and 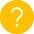 unclear.

A formal tool of bias assessment, ROBINS-I, was used to assess the risk of bias in the non-randomized studies of interventions included in this review (Sterne *et al.*, 2016). Classification of interventions obtained the highest risk of bias, enabling us to determine that most of the studies (80%) were inadequate in terms of setting, frequency or chemotherapy dose. This is because, for an appropriate comparison of interventions—as vincristine is given in combination treatments—the interventions need to be well defined. Conversely, selection bias conferred the lowest risk (60% of studies), with bias introduced when patients who were re-treated with chemotherapy following a relapse were maintained in the cohort, which would normally be regarded as ineligible for inclusion. Moreover, the risk of bias of reported results was calculated as unclear in 75% of studies. Information of the quality assessment for the studies that included either a vincristine sub-analysis or a control group that did not receive vincristine can be found in Fig. 2, and extensive reasons for the level of bias assigned across all 24 eligible studies are included in Supplementary Table SIV.

### Fertility and parenthood

Of the five publications that included pregnancy and fertility outcomes of childhood cancer survivors (CCS), only two involved vincristine sub-analysis and/or a control group (Table II). This included the multi-centre cohort Childhood Cancer Survivor study (CCSS) (Green *et al.*, 2010), which comprised 6224 male survivors. Cancer survivors were found less likely to sire a pregnancy when compared to their siblings (hazard ratio (HR) = 0.56). The HR of siring a pregnancy with an alkylating-agent dose (AAD) score of 0, and a radiation dose to the hypothalamus/pituitary and testes of 0, was 0.91 (95% CI, 0.73–1.14; *P* = 0.41), indicating that alkylating agent chemotherapeutics and/or radiotherapy are important contributors to reduced fertility (Green *et al.*, 2010). As illustrated in continual multivariable models, the effect of vincristine and vinblastine incorporation into AAD resulted in an HR for a chance of partner pregnancy of 1.07 among male survivors for vincristine (95% CI, 0.87–1.32; *P* = 0.53) and 1.11 for vinblastine (95% CI, 0.59–2.08; *P* = 0.75). Similar results were obtained when considering the CED, where the HR for partner pregnancy was 1.04 for vincristine (95% CI, 0.85–1.28; *P* = 0.69) and 1.00 for vinblastine (95% CI, 0.60–1.67; *P* = 0.99) (Green *et al.*, 2014). Together, these results suggest that vinca alkaloids do not significantly contribute to gonadotoxicity in childhood cancer (Green *et al.*, 2014).

**Table II:** Summary of studies with vincristine sub-analysis in survivors of childhood cancer exposed to vincristine-containing regimens and the outcome measure of partner pregnancy and fertility.

Study	Diagnoses	Age at diagnosis (years)	Male survivors (n)	Prepubertal survivors (n)	Chemotherapy treatment	Control group	Vincristine analysis	Summary finding
Green *et al.* (2014)	Various	5–20	4579	NS	Various	Yes^a^	Yes^b^	Chemotherapeutics demonstrated a significant association between CED and chance of siring a pregnancy in the multivariate model; Similar rate of fatherhood after vincristine compared to no vincristine. Statistical data provided.
Green *et al.* (2010)	Various	0–20	6224	NS	Various	Yes^a^	Yes^b^	Chemotherapeutics demonstrated a significant association between AAD and chance of siring a pregnancy in the multivariate model; Similar rate of fatherhood after vincristine compared to no vincristine. Statistical data provided.

AAD, alkylating agent dose; CED, cyclophosphamide equivalent dose; HR, hazard ratio; NS, not Specified.

a Healthy siblings.

b Multivariate and univariate analyses.

Further studies obtained from our literature search did not include an individual sub-analysis of the contribution of vinca alkaloids to pregnancy or fertility rates, or appropriate control groups for deciphering vincristine effects, therefore these studies are not further discussed (Green *et al*, 1989; Hamre *et al.*, 2012; Shiraishi and Matsuyama, 2014).

### Semen analysis

Sixteen studies were identified that described the effect of vincristine-containing chemotherapy on the subsequent adulthood sperm count of childhood survivors (Table I). Cut-offs for semen analysis varied across studies (Supplementary Table SIII): not all were based on World Health Organization (WHO) standards. Two of these studies included a vincristine sub-analysis and one additional study had a control group of patients that had not received vincristine (Table III).

**Table III:** Summary of studies with vincristine sub-analysis and/or control group in survivors of childhood cancer exposed to vincristine-containing regimens and the outcome measure of semen analysis.

Study	Diagnoses	Age at diagnosis (years)	Male survivors (n)	Prepubertal survivors (n)	Chemotherapy treatment	Cranial/infradiaphragmatic irradiation	Control group	Vincristine analysis	Summary finding
Kruseová *et al.* (2021) ^a^	Various	0.1–19.1	143	66	Various	Yes (n = 34)	Yes	Yes^c^	65% of the total survivors had abnormal semen quality. No significant risk illustrated with the administration of topoisomerase and mitotic inhibitors.^d^
Beaud *et al.* (2019) ^a^	Various	7.4–11	13	6	Vinca alkaloids; alkylating agents; anthracyclines	No	Yes^b^	Yes^c^	Non-significant correlation for vinca alkaloid cumulative doses when concerned with DFI and sperm count, illustrating no vinca alkaloid effect.^d^
Müller *et al.* (1996)	Hodgkin’s disease	3–17	33	13	Various	Yes	Yes^b^	No	Therapy with alkylating agents and/or gonadal radiation were risk factors for development of azoospermia.

DFI, DNA fragmentation index.

a Semen analysis based on World Health Organization laboratory guidelines, for processing and examination of human semen.

b Healthy age and sex-matched controls.

c Sub-group analysis for vincristine/vinca alkaloid/topoisomerase inhibitors in childhood survivors.

d Summary findings relate to chemotherapy treatment received by prepubertal patients (≤12 years of age).

Semen quality was assessed in a cohort of 143 long-term CCS, of whom 66 were prepubertal patients with a median age of 7.76 years (range, 0.12–12.56) at cancer diagnosis (Kruseová *et al.*, 2021). This study found abnormal semen analysis results for 65% of the 143 (including pre- and post-pubertal) survivors, compared to 26.5% of healthy controls (*P* < 0.0001) and confirmed that the highest risk for semen abnormalities was in the survivors treated with alkylating agents (odds ratio (OR) = 3.595; *P* < 0.008). Although risks for abnormal semen analyses were found with the administration of other drugs, no significant difference was illustrated for the group consisting of topoisomerases and mitotic inhibitors (which includes vinca alkaloids) during sub-analysis. For this group, abnormal semen analysis was identified in 64% of the patients, although the risk was not significantly increased compared to those who did not receive these agents (OR = 2.270; *P* = 0.499) (Kruseová *et al.*, 2021).

A study of 13 CCS, of whom three were prepubertal at diagnosis, reported a prevalence of 38% for low sperm count, with 5/13 samples having sperm concentrations below the WHO standards. Of those patients who were reported to be prepubertal at diagnosis, 3/6 (50%) had a reduced sperm count. Three prepubertal patients were reported to have received vincristine. Of these 2/3 that had also received alkylating agents had abnormal sperm counts (0 and 2.4 million sperm), whilst the patient who had received vincristine without alkylators had a normal sperm count (854.7 million sperm). Overall, no significant correlation was reported for vinca alkaloid cumulative doses and either sperm DNA fragmentation index (*r*_s_ = 0.16, *P* > 0.05) or sperm count (*r*_s_ = 0.07, *P* > 0.05).

Additionally, one study compared semen analysis in CCS receiving regimens that contained vincristine with a healthy control group (Müller *et al.*, 1996). In this study comparing 33 CCS (age range, 3–17 years at diagnosis) with healthy age-matched controls, 9/14 (64%) of the CCS who could be assessed by spermiogram were azoospermic, whereas 8/8 (100%) of the controls were normozoospermic. Azoospermic CCS (n = 9) were more often treated with alkylating agents and higher gonadal doses of radiation (*P* < 0.05) than were the normozoospermic CCS. Whilst cumulative vincristine doses were calculated for the CCS in this study, the specific impact of vincristine on semen analysis was not reported (Müller *et al.*, 1996).

The remainder of the included studies did not specifically report the contribution of vincristine to semen parameters and did not include healthy controls for comparative purposes. Extensive information of these studies can be found in Supplementary Table SIII.

### Gonadotrophins, testicular volumes and puberty onset

Nineteen studies were identified that described gonadotrophins, testicular volume or puberty in CCS who had received vincristine-based chemotherapy (Table I), with six of these studies including a control group of patients that had not received vincristine (Table IV).

**Table IV:** Summary of studies with vincristine sub-analysis and/or control group in survivors of childhood cancer exposed to vincristine-containing regimens and the outcome measure of gonadotrophins, testicular volume and puberty.

Study	Diagnoses	Age at diagnosis (years)	Male survivors (n)	Prepubertal survivors (n)	Chemotherapy treatment	Cranial/infradiaphragmatic irradiation	Control group	Vincristine analysis	Summary findings
Utriainen *et al.* (2019)	Neuroblastoma	0.2–3.6	9	NS	Multiple regimens	No	Yes^a^	No	Gonadal failure manifestation with elevated FSH levels with the need of androgen substitution and atrophic testes in 4/6 male survivors. Specific impact of vincristine not investigated.
Krawczuk-Rybak *et al.* (2009)	Acute lymphoblastic leukaemia	6.2–10.6	59	51	Multiple regimens	Yes (n = 26)	Yes^a^	No	Inhibin B-to-FSH ratio reduced; testicular volume was evidently lower when compared to healthy controls.
Beaud *et al.* (2019) ^a^	Various	7.4–11	13	6	Vinca alkaloids; alkylating agents; anthracyclines	No	Yes^a^	No	FSH levels >10 IIU/l (suggestive of azoospermia) in 3/6 childhood cancer survivors, compared to 0/12 controls.
Kruseová *et al.* (2021)	Various	0.1–19.1	143	66	Multiple regimens	Yes (n = 34)	Yes	No^b^	Increased FSH/LH levels with time since diagnosis; survivors treated with alkylating agents were more likely to have increased gonadotrophin levels. No impacts attributed to vincristine.
Müller *et al.* (1996)	Hodgkin’s disease	3–17	33	NS	Multiple regimens	Yes	Yes^a^	No	Reduced testicular volume and raised FSH levels in patients compared with healthy controls.
Dhabhar *et al.* (1993)	Hodgkin’s disease	4–15	26	23	COPP, MOPP, ABVD	No	Yes^a^	No	Hormonal analysis showed elevated mean levels of FSH and inhibin, whereas LH was only marginally elevated.

COPP, Cyclophosphamide, Oncovin (brand name for vincristine), Methotrexate and Prednisone; MOPP, Mustargen (brand name for mechlorethamine), Oncovin, Procarbazine and Prednisone; ABVD, Adriamycin, Bleomycin, Vinblastine, Dacarbazine (similar to procarbazine but less gonadotoxic, designated as P in COPP and MOPP).

a Healthy age and sex-matched controls.

b Topoisomerase and mitotic inhibitor sub-analysis performed for semen analysis only.

One of the studies illustrated that after a median follow-up of 19 years, 6/9 of survivors fulfilled the criteria of gonadal failure owing to absent puberty with a need for androgen substitution, low total (bilateral) testicular volumes (8.5 versus 39 ml, *P* < 0.001) or elevated FSH levels (26.3 versus 3.7 IU/l, *P* < 0.001), when compared to age-matched controls (Utriainen *et al.*, 2019). Patients in this study might have received different vincristine induction chemotherapy; however, specific treatment descriptions were not available for individual patients.

In a cohort of 59 (51 prepubertal) male CCS treated for ALL, all of whom had received vincristine, there was a reduced testicular volume (14.8 ± 5.1 versus 18.5 ± 4.7 ml, respectively; *P* = 0.01) at puberty, compared to healthy controls. In addition, a small number of patients displayed evidence of raised FSH at puberty. However, no difference was reported in mean values of FSH, LH, testicular volume or testosterone between the pubertal patients who had been treated in prepuberty and healthy pubertal controls. Despite this, inhibin B, an indirect indicator of spermatogenesis, was lower at puberty in patients treated during prepuberty, compared to healthy pubertal controls (Krawczuk-Rybak *et al.*, 2009).

The study by Beaud *et al.* (2019) reported FSH levels in adult patients who had received vincristine-containing treatment regimens prior to puberty. Raised FSH (>10 IU/l) was demonstrated in 3/6 patients, compared with 0/12 healthy controls. Whilst cumulative vinca alkaloid exposure had been determined based on semen analysis, this vincristine exposure was not reported for hormone analysis (Beaud *et al.*, 2019).

Kruseová *et al.* (2021) reported a positive correlation between elevated levels of FSH and abnormal semen analysis. Whilst this study had shown no association between topoisomerase/mitotic inhibitor exposure and semen analysis, no such sub-analysis was reported for gonadotrophins.

In the study by Müller *et al.* (1996), FSH was significantly increased in CCS, whilst testicular volume was significantly decreased, compared with healthy controls. As previously mentioned in relation to semen analysis, whilst cumulative vincristine doses were calculated for the CCS in this study, the specific impact of vincristine on semen analysis was not reported (Müller *et al.*, 1996).

In a study of 26 CCS with Hodgkin’s disease, all of whom had received vincristine-containing regimens, 23 had gonadotrophins assessed in adulthood. Overall, FSH was significantly increased in the patients, compared with the healthy controls. These patients had all received significant cumulative exposure to alkylating agents and therefore the relative contribution of vincristine to gonadotoxicity cannot be determined (Dhabhar *et al.*, 1993).

The remainder of the included studies did not specifically report the contribution of vincristine to gonadotrophins, testicular volume and puberty parameters and did not include healthy controls for comparative purposes. Extensive information of these studies can be found in Supplementary Table SIII.

### Histological evidence for testicular development and function

Although the histological analysis was not specifically defined as a primary outcome measure, we identified several studies that described the impact of regimens that included vincristine on prepubertal testicular tissues (Table V). A cohort including 24 CCS treated for ALL had testicular sections performed and analysed to determine the morphological alterations post-chemotherapy (Anelli *et al.*, 1984). Most of the patients were treated with a combination of vincristine, prednisone, l-asparaginase, 6-mercaptopurine and methotrexate; none of which are considered to produce prolonged effects on the male gonads. More specifically, the numbers of spermatogonia per tubule and tubular diameter were unaffected in the age Group I, 2.5–5 years, but were reduced in Group II, 6–9 years, when compared with control testis of the same age, which could lead to subsequent effects on fertility owing to spermatogonial degeneration or mitotic arrest in the existing spermatogonial population. However, the control values and percentage decline were not given as part of the narrative of this research, and the difference between Groups I and II did not reach statistical significance (*P* < 0.08) (Anelli *et al.*, 1984). In another study of 23 CCS diagnosed at a mean age of 5.7 ± 2.9 years (Nurmio *et al.*, 2009), immunohistochemistry was performed for the expression of spermatogonial markers, to be compared with gonadal function after sexual maturation in adulthood. Several MAGE-A4, CD9 or OCT4-positive germ cells were identified after standard risk treatment without cyclophosphamide (Nurmio *et al.*, 2009). Nonetheless, both studies did not distinguish between the impacts attributed to vincristine or other agents.

**Table V:** Summary of histological studies in survivors of childhood cancer exposed to vincristine-containing regimens.

Study	Diagnoses	Age at diagnosis (years)	Male survivors (n)	Prepubertal survivors (n)	Chemotherapy treatment	Cranial/infradiaphragmatic irradiation	Control group	Vincristine analysis	Summary findings
Nurmio *et al.* (2009)	Acute lymphoblastic leukaemia	5.7 ± 2.9*	23	23	Multiple regimens	Yes (n = 4)	No	No	50% reduction in spermatogonial number between induction (10 mg/m^2^ vincristine) and end of standard risk treatment (18 mg/m^2^ vincristine cumulative). TV, FSH, LH, testosterone and inhibin B in adulthood were similar between CCS and a reference population of healthy men.
Anelli *et al.* (1984)	Acute lymphoblastic leukaemia	2–13	24	21	Multiple regimens	Yes (n = 1)	No	No	Spermatogonia per tubule were unaffected in those aged 2.5–5 years but were reduced in those 6–9 years, compared with age-matched control testis.
Medrano *et al.* (2021)	Various	0–16	28	NS	Multiple regimens	NS	Yes^a^	Yes	No significant correlation between cumulative vincristine dose and testicular phenotype as assessed by elastic net logistic regression.

NS, not specified; TV, testicular volume; CCS: childhood cancer survivors.

a Age-matched controls. *Mean ± SEM

A recent study evaluated the impact of individual treatments on testicular histologic phenotype, in paediatric patients (n = 28) aged 0–16 years when compared with age-matched controls (Medrano *et al.*, 2021). Vincristine cumulative doses were recorded for the weakly affected (n = 9) (mean, 5.83 ± 9.55 mg/m^2^) and the severely affected patients (n = 19) (mean, 11.10 ± 12.41 mg/m^2^). However, the statistic model did not find a significant correlation between vincristine dose and the testicular phenotype, as only the drugs that were selected by the elastic net model were taken forward into the Bayesian regression model (Medrano *et al.*, 2021).

## Discussion

### Summary of evidence

To the best of our knowledge, this is the first systematic review to investigate the published data on long-term fertility outcomes of CCS that were treated with vincristine-containing chemotherapy. Fertility is often compromised following chemotherapy treatment, with outcomes traditionally involving a reduced chance of siring a pregnancy, decreased sperm counts and elevated levels of gonadotrophins. Owing to the high variability of findings across papers, it was not possible to carry out a meta-analysis in this systematic review and thus a qualitative approach to data analysis was performed.

Collected data examined the impact of chemotherapy on partner pregnancy and provided evidence for the specific effects of vincristine in some of the studies. One large study presenting results from the CCSS demonstrated that when confounding sources of cumulative alkylating-agent exposure or radiation were accounted for, the chemotherapy treatment without such sources was less damaging to the testicular tissue and there was a greater likelihood of partner pregnancy (Green *et al.*, 2010). Green *et al.* (2010) also excluded pregnancies that resulted from ART during analysis, increasing validity in terms of natural conceptions as an obtained outcome. In accordance, multivariate analysis in another study involving the CCSS cohort also reported that vincristine exposure is not a significant risk factor for siring a pregnancy (Green *et al.*, 2014). Both studies possess homogeneity of strength, which arises because both involve the CCSS cohort, the largest and most-characterized cohort of survivors of childhood cancer. This allows interrogation of the relation between drug exposure and subsequent effects on fertility with increased statistical power when compared with other cohorts.

Semen analysis represents an objective and precise measure of male fertility potential and could therefore be less inclined to bias than pregnancy rates, which are heavily influenced by social factors and cannot confirm paternity. Kruseová *et al.* (2021) specifically addressed the effects of mitotic inhibitors, with no significant association found for abnormal semen analyses. This cohort excluded patients with subsequent neoplasms and further treatment with chemotherapy. However, the evaluated semen analysis was mainly conducted in expected high-risk Hodgkin’s lymphoma CCS with a higher degree of fertility problems. In addition, Beaud *et al.* (2019) reported a very weak monotonic correlation between cumulative doses of vinca alkaloids and sperm count. However, the fact that this was not statistically significant does not support a conclusion of an effect of these agents on sperm counts.

Additionally, the finding of persistent azoospermia in men after chemotherapy administration including vincristine is consistent with findings of morphological alterations and tubule disruption (Anelli *et al.*, 1984). Nurmio e*t al.* (2009) further illustrate damage to the spermatogonial stem cell population after cancer treatment, which is also considered the likely target of damage during childhood chemotherapy. Nonetheless, the results of germ cell loss from histological examination of testicular biopsies are confounded by alkylating-agent cumulative doses and irradiation exposure. The individual contribution of vincristine to germ cell loss cannot be concluded from these studies. However, vinca alkaloids were described in the past as less damaging than cytarabine and cyclophosphamide. These studies were based on single-agent effects on the seminiferous tubules of the adult rodent (Lu and Meistrich, 1979). The data of Nurmio *et al.* (2009) show that the effects of induction therapy (10 mg/m^2^ vincristine, asparaginase and prednisolone) reduce spermatogonial numbers by about 50%, but subsequent high-dose therapy reduces these numbers more dramatically. This suggests that, at most, mild effects may occur in human testis at this vincristine dose (10 mg/m^2^), when compared to the more severe effects seen after vincristine exposure (6 mg/m^2^) in the mouse study by Delessard *et al.* (2020). Furthermore, a recent study involving analysis of prepubertal testis biopsies obtained from CCS did not identify a change in the percentage of tissue cross-sections with proliferating spermatogonia based on exposure to topoisomerase inhibitors (Medrano *et al.*, 2021). In addition, although there seems to be a trend for vincristine and testicular phenotype, the statistic model did not find a significant correlation in this study. However, it remains possible that prepubertal exposure to vinca alkaloids does affect testicular function, given the limited sample size, number of variables studied and diverse range of treatment regimens received by patients (Medrano *et al.*, 2021).

Gonadotrophins provide an indirect assessment of spermatogenesis and testicular function. Elevated levels indicate primary hypogonadism post-therapy, and an FSH threshold can be used as a proxy for identifying impaired spermatogenesis in survivors during adulthood (Kelsey *et al.*, 2017). Predicting azoospermia via gonadotrophin levels in prepubertal patients is not possible as the hypothalamic–pituitary–gonadal axis in prepuberty is relatively quiescent. Moreover, the specific contribution of vincristine was only assessed in one of six studies with reported gonadotrophin data. Kruseová *et al.* (2021) demonstrated a positive correlation between the elevated FSH levels and abnormal semen analysis for the majority of the cohort; however, these outcomes were not attributed to vincristine after sub-analysis (Kruseová *et al.*, 2021).

### Strengths and limitations

The strengths of this systematic review include the comprehensive nature of the search strategy, including publications identified through bibliographic screens of reviews. Given the heterogeneous nature of the studies and risk of bias, quality assessment of the non-randomized studies is also important. Selection bias was reduced by prior registration of the review before the initial search, and an effort to further reduce potential error in the selection of studies was achieved via the cooperation of two assessors throughout the methodology process.

Whilst the results of this review do not support the hypothesis that exposure to vinca alkaloids during prepuberty significantly impacts future testicular function and fertility, there are important limitations that must be considered. These primarily relate to the availability of data that permit interrogation of the specific contribution of vincristine. In most cases, comparator regimens without vincristine were not available and single-agent vincristine is only used in the paediatric population for the treatment of haemangiomas, with no long-term follow-up of fertility outcomes reported. It is possible that vincristine could have synergistic effects on fertility when used as part of multi-agent treatment regimens. Despite this, it is known that the majority of survivors of ALL are fertile on long-term follow-up, and since as a patient cohort they receive the highest cumulative dose of vincristine during their treatment, this would support the overall finding of this review. Furthermore, only a few studies conducted a vincristine sub-analysis to better characterize its relative contribution to fertility outcomes and nearly all had a small sample size, further reducing the power of results obtained and increasing the potential margin of error. In addition, several studies were not completely characterized in terms of setting and frequency or dose of chemotherapy exposure.

### Evidence for effects of vincristine on fertility in rodent studies

*In vitro* studies using spermatogonial cell lines with stem cell characteristics have shown that exposure to vincristine can result in increased apoptosis and cell death (Wyns *et al.*, 2010; Beaud *et al.*, 2017). In addition, an *in vivo* approach was recently explored to expose prepubertal rodents to vincristine at doses currently considered as low-gonadotoxicity in humans (Delessard *et al.*, 2020). Exposure of prepubertal mice to vincristine (200 µg/kg) before the initiation of the first wave of spermatogenesis resulted in morphological alterations of the testicular tissue over the short and long term, resulted in a reduction in the number and quality of epididymal sperm, and reduced the fertility (>90% reduction in birth rate) of adult male mice, compared to untreated control males (Delessard *et al.*, 2020). Administration of such a drug as part of induction or consolidation chemotherapy for paediatric cancers could therefore imply adverse effects on the fertility of survivors who have not yet benefitted from a fertility preservation procedure.

### Future studies to determine the impact of vinca alkaloids on fertility in males

The results of the animal-based studies are in contrast to the findings of this review of the clinical data and the current gonadotoxic classification risk for vincristine in humans. Consequently, future research should focus on large-scale prospective studies (including patient registries) in CCS in order to accurately correlate the impact of vincristine with the clinical and biochemical assessment of future fertility. In addition, experimental studies using human-relevant systems can determine the relative gonadotoxicity of vincristine, either alone or in combination with other agents, and also allow the mechanisms involved in gonadotoxicity to be investigated (Baert *et al.*, 2020; Tharmalingam *et al.*, 2020). Given its reported gonadotoxicity in rodents, this review focused on the effects of vincristine. However, vinblastine is also used for central nervous system tumours, particularly low-grade glioma, and given its use as a single-agent treatment it may also be possible to determine the gonadotoxicity of this particular vinca alkaloid.

## Conclusion

Cancer is the second leading cause of death in childhood, and it is approximated that 1 in 530 young adults is a survivor. However, the impacts of cancer treatment during childhood on future fertility in males are yet to be fully determined. Overall, the evidence presented in this review did not support the hypothesis that exposure to vincristine negatively impacts future fertility in males. However, there are important limitations to the existing studies including confounding by multiple treatment regimens, study size and limited cohorts with accurate long-term fertility outcomes. Future research should be aimed at conducting experimental studies using human-relevant model systems and large-scale prospective clinical cohort studies to accurately predict the effect of vinca alkaloid (and other exposures) on testicular function and fertility, in order to ensure optimal counselling and provision of fertility preservation options to young males with cancer.

## Supplementary Material

dmac039_Supplementary_Data

## Data Availability

The data underlying this article are available in the article and in its Supplementary Material.
