# Supplementary material for: The impact of vincristine on testicular development and function in childhood cancer
Source: Hum Reprod Update. 2022 Dec 10;29(2):233–45. doi: 10.1093/humupd/dmac039 (PMC9976970; doi:10.1093/humupd/dmac039)
Supplement: dmac039_Supplementary_Data [file dmac039_supplementary_data.docx]

**Supplementary Table SI** Methodology and search strategy used for identification of publications relating to testicular development and function after vincristine chemotherapy in childhood cancer survivors. Search strategy adapted from Tian En et al. (2020).

**Vincristine Search Strategy on Scopus**

(TITLE-ABS("vincristine" OR "leurocristine" OR "Oncovin" OR "Vincristine Sul*ate" OR "leurocristine sul*ate" OR "LCR" OR "VCR" OR "Vincasar PFS" OR "Vincosid" OR "Vincrex" OR "Kyocristine" OR "Vincasar" OR "Marqibo" OR "alkylating" OR "alkylating agent" OR "alkylating agents" OR "antineoplastic alkylating agents" OR "R-CHOP")) AND (TITLE-ABS-KEY("male" OR "males" OR "boy" OR "boys" OR "boyfriend" OR "boyhood")) AND (TITLE-ABS-KEY(“child” OR “children” OR "prepubertal" OR "prepubertal testis" OR "prepuberty" OR "peripuberty" OR "puberty" OR "childhood cancer" OR "children's cancer")) AND (TITLE-ABS-KEY("leuk*mia" OR "acute leuk*mia" OR "acute myeoloid leuk*mia" OR "acute lymphoblastic leuk*mia" OR "Hodgkin's" OR "Hodgkin's Disease" OR "Hodgkin's lymphoma" OR "Hodgkin" OR "non-Hodgkin" OR "non-Hodgkin's" OR "non- Hodgkin's lymphoma" OR "neuroblastoma" OR "neuroblastoma*" OR "rhabdomyosarcoma" OR "rhabdomyosarcomas" OR "Ewing's sarcoma" OR "Ewing" OR "Ewing*" OR "Wilms' tum*r" OR "nephroblastoma" OR "multiple myeloma" OR "chronic leuk*mia" OR "chronic leuk*mias" OR "thyroid cancer" OR "brain tum*r" OR "Small Cell Lung" OR "Small Cell Lung Cancer" OR "p*diatric oncology" OR "childhood cancer" OR "childhood tum*r" OR "childhood tum*rs" OR "brain neoplasms" OR "central nervous system neoplasm" OR "central nervous system neoplasms" OR "central nervous system tum*r" OR "central nervous system tum*rs" OR "brain cancer*" OR "brain neoplasm*" OR "intracranial neoplasm*" OR "testis neoplasm" OR "testicular neoplasm" OR "testicular neoplasms" OR "testis cancer" OR "testicular cancer" OR "testis tum*r" OR "testicular cancer" OR "cancer of testis" OR "testis neoplasm*" OR "testis tum*r" OR "testis tum*rs" OR "lymphocytic" OR "acute")) AND (TITLE-ABS("spermatogenesis" OR "spermiogenesis" OR "spermatocytogenesis" OR "spermatogenic failure" OR "azoospermia" OR "oligospermia" OR "asthenozoospermia" OR "teratozoospermia" OR "oligoasthenoteratozoospermia" OR "dysspermia" OR "normozoospermic" OR "gonadal disorder" OR "semen" OR "semen analysis" OR "semen quality" OR "sperm" OR "sperm count" OR "sperm motility" OR "spermatozoa" OR "progeny" OR "offspring" OR "fertility" OR "infertility" OR "subfertility" OR "reproduction" OR "fertili*ation" OR "conception" OR "paternity" OR "fatherhood" OR "parenthood" OR "pregnancy outcome" OR "fertile" OR "infertile" OR "subfertile" OR "sperm maturation" OR "aspermia" OR "spermatozoon abnormality" OR "germ cell" OR "spermatogonia" OR "spermatogonial" OR "spermatogonium" OR "meiosis" OR "gonocyte" OR "spermatid" OR "spermatids" OR "follicle stimulating hormone" OR "FSH" OR "sertoli cells" OR "testicular development" OR "testicular function" OR "testicle development" OR " testicle function" OR "testis"))

**Vincristine Search Strategy on PubMed**

(((("Vincristine"[Mesh] OR "vincristine" OR "leurocristine" OR "Oncovin" OR "Vincristine Sulfate" OR "leurocristine sulfate" OR "Vincristine Sulphate" OR "LCR" OR "VCR" OR "Marqibo" OR "alkylating" OR "alkylating agent" OR "alkylating agents" OR "antineoplastic alkylating agents" OR "R-CHOP") AND (male[tiab] OR "males" OR "boy" OR "boys" OR "boyfriend" OR "boyhood")) AND ("child" OR "children" OR "prepubertal" OR "prepubertal testis" OR "prepuberty" OR "peripuberty" OR "puberty" OR "childhood cancer" OR "children’s cancer")) AND ("leukemia" OR "leukaemia" OR "acute leukemia" OR "acute leukaemia" OR "acute lymphoblastic leukemia" OR "acute lymphoblastic leukaemia" OR "Hodgkin's" OR "Hodgkin’s Disease" OR "Hodgkin's lymphoma" OR "Hodgkin" OR "non-Hodgkin" OR "non-Hodgkin's" OR "non- Hodgkin's lymphoma" OR "neuroblastoma" OR "neuroblastomas" OR "rhabdomyosarcoma" OR "rhabdomyosarcomas" OR "Ewing's sarcoma" OR "Ewing" OR "Ewing*" OR "Wilms' tumor" OR "Wilms' tumour" OR "nephroblastoma" OR "multiple myeloma" OR "chronic leukemia" OR "chronic leukaemia" OR "chronic leukemias" OR "chronic leukaemias" OR "thyroid cancer" OR "brain tumor" OR "brain tumour" OR "Small Cell Lung" OR "Small Cell Lung Cancer" OR "pediatric oncology" OR "paediatric oncology" OR "childhood cancer" OR "childhood tumor" OR "childhood tumors" OR "childhood tumour" OR "childhood tumours" OR "brain neoplasms" OR "central nervous system neoplasm" OR "central nervous system neoplasms" OR "central nervous system tumor" OR "central nervous system tumors" OR "central nervous system tumour" OR "central nervous system tumours" OR "brain cancer*" OR "brain neoplasm*" OR "intracranial neoplasm*" OR "testis neoplasm" OR "testicular neoplasm" OR "testicular neoplasms" OR "testis cancer" OR "testicular cancer" OR "testis tumor" OR "testis tumour" OR "testicular cancer" OR "cancer of testis" OR "testis neoplasm*" OR "testis tumor" OR "testis tumors" OR "testis tumour" OR "testis tumours" OR "lymphocytic" OR "acute")) AND ("Spermatogenesis"[Mesh] OR "spermatogenesis" OR "spermiogenesis" OR "spermatocytogenesis" OR "spermatogenic failure" OR "azoospermia" OR "oligospermia" OR "asthenozoospermia" OR "teratozoospermia" OR "oligoasthenoteratozoospermia" OR "dysspermia" OR "normozoospermic" OR "gonadal disorder" OR "semen" OR "semen analysis" OR "semen quality" OR "sperm" OR "sperm count" OR "sperm motility" OR "spermatozoa" OR "progeny" OR "offspring" OR "fertility" OR "infertility" OR "subfertility" OR "reproduction" OR "fertilisation" OR "fertilization" OR "conception" OR "paternity" OR "fatherhood" OR "parenthood" OR "pregnancy outcome" OR "fertile" OR "infertile" OR "subfertile" OR "sperm maturation" OR "aspermia" OR "germ cell" OR "spermatogonia" OR "spermatogonial" OR "spermatogonium" OR "meiosis" OR "gonocyte" OR "spermatid" OR "spermatids" OR "follicle stimulating hormone" OR "FSH" OR "sertoli cells" OR "testicular development" OR "testicular function" OR "testis development" OR " testis function" OR "testis")

**Supplementary Table SII** Publications that underwent a detailed full-text screening initially, before being excluded due to not meeting at least one aspect of inclusion criteria.

| **Author(s)** | **Year** | **Title** | **Journal** | **Volume (Issue)** | **Pages** | **DOI** | **Reason(s) for Exclusion** |
| --- | --- | --- | --- | --- | --- | --- | --- |
| de Rezende ACP, Weltman E, Chen MJ, Helito JK, de Carvalho ÍT, Sakuraba RK, Silva NS, Cappellano AM, Hamerschlak N. | 2019 | Intensity-modulated ventricular irradiation for intracranial germ-cell tumors: Survival analysis and impact of salvage re-irradiation. | PLoS One | 14 (12) | e0226350 | <https://doi.org/10.1371/journal.pone.0226350> | Vincristine not part of chemotherapy protocols. |
| Van Iersel L., Li Z., Srivastava D.K., Brinkman T.M., Bjornard K.L., Wilson C.L., Green D.M., Merchant T.E., Pui C.-H., Howell R.M., Smith S.A., Armstrong G.T., Hudson M.M., Robison L.L., Ness K.K., Gajjar A., Krull K.R., Sklar C.A., Van Santen H.M., Chemaitilly W. | 2019 | Hypothalamic-Pituitary Disorders in Childhood Cancer Survivors: Prevalence, Risk Factors and Long-Term Health Outcomes. | Journal of Clinical Endocrinology and Metabolism | 104 (12) | 6101-6115 | <https://doi.org/10.1210/jc.2019-00834> | Non-relevant chemotherapy concentrated study with effects on fertility. |
| Stukenborg JB, Alves-Lopes JP, Kurek M, Albalushi H, Reda A, Keros V, Töhönen V, Bjarnason R, Romerius P, Sundin M, Norén Nyström U, Langenskiöld C, Vogt H, Henningsohn L, Mitchell RT, Söder O, Petersen C, Jahnukainen K. | 2018 | Spermatogonial quantity in human prepubertal testicular tissue collected for fertility preservation prior to potentially sterilizing therapy. | Hum Reproduction | 33 (9) | 1677-1683 | <https://doi.org/10.1093/humrep/dey240> | No reference of vincristine/vinca- alkaloids. |
| Green DM, Zhu L, Wang M, Chemaitilly W, Srivastava D, Kutteh WH, Ke RW, Sklar CA, Pui CH, Kun LE, Ribeiro RC, Robison LL, Hudson MM. | 2017 | Effect of cranial irradiation on sperm concentration of adult survivors of childhood acute lymphoblastic leukemia: a report from the St. Jude Lifetime Cohort Study† | Human Reproduction | 32 (6) | 1192-1201 | <https://doi.org/10.1093/humrep/dex082> | Vincristine not part of chemotherapy regimen. |
| Boltežar L., Pintarić K., Jezeršek Novaković B. | 2016 | Fertility in young patients following treatment for Hodgkin’s lymphoma: a single center survey. | Journal of Assisted Reproduction and Genetics | 33 (3) | 325-333 | <https://doi.org/10.1007/s10815-015-0636-6> | Receiving therapy in adulthood. |
| Gunn Harriet M., Rinne I.,  Emilsson H., Gabriel M.,  Maguire Ann M., Steinbeck Katharine S. | 2016 | Primary Gonadal Insufficiency in Male and Female Childhood  Cancer Survivors in a Long-Term Follow-Up Clinic. | Journal of Adolescent and Young Adult Oncology | 5 (4) | 344-350 | https://doi.org/10.1089*/*jayao*.*2016.0007 | Vincristine not part of chemotherapy regimen. |
| Nieto Y, Tu SM, Bassett R, Jones RB, Gulbis AM, Tannir N, Kingham A, Ledesma C, Margolin K, Holmberg L, Champlin R, Pagliaro L. | 2015 | Bevacizumab/high-dose chemotherapy with autologous stem-cell transplant for poor-risk relapsed or refractory germ-cell tumors. | Annals of Oncology | 26 (10) | 2125-2132 | <https://doi.org/10.1093/annonc/mdv310> | Vincristine not part of chemotherapy regimen. |
| Panasiuk A, Nussey S, Veys P, Amrolia P, Rao K, Krawczuk-Rybak M, Leiper A. | 2015 | Gonadal function and fertility after stem cell transplantation in childhood: comparison of a reduced intensity conditioning regimen containing melphalan with a myeloablative regimen containing busulfan. | British Journal of Haematology | 170 (5) | 719-726 | <https://doi.org/10.1111/bjh.13497> | Vincristine not part of chemotherapy regimen. |
| No authors listed | 2015 | Treatment of childhood cancers: late effects. | Prescrire International | 24(164) | 236-239 | DOI unavailable | Only abstract could be found. |
| Wilhelmsson M, Vatanen A, Borgström B, Gustafsson B, Taskinen M, Saarinen-Pihkala UM, Winiarski J, Jahnukainen K. | 2014 | Adult testicular volume predicts spermatogenetic recovery after allogeneic HSCT in childhood and adolescence. | Pediatric Blood Cancer | 61 (6) | 1094-1100 | <https://doi.org/10.1002/pbc.24970> | Vincristine not part of chemotherapy regimen. |
| Miller SD, Li Y, Meyers KE, Caplan A, Miller VA, Ginsberg JP. | 2014 | Fertility preservation in paediatric nephrology: results of a physician survey. | Journal of Renal Care | 40 (4) | 257-262 | <https://doi.org/10.1111/jorc.12079> | No fertility outcomes. |
| Winther J.F., Olsen J.H., Wu H., Shyr Y., Mulvihill J.J., Stovall M., Nielsen A., Schmiegelow M., Boice Jr. J.D. | 2012 | Genetic Disease in the Children of Danish Survivors of  Childhood and Adolescent Cancer. | Journal of Clinical Oncology | 30 (1) | 27-33 | <https://doi.org/10.1200/JCO.2011.35.0504> | Non-relevant chemotherapy concentrated study with effects on fertility. |
| Van Der Kaaij M.A.E., Heutte N., Meijnders P., Abeilard-Lemoisson E., Spina M., Moser L.C., Allgeier A., Meulemans B., Dubois B., Simons A.H.M., Lugtenburg P.J., Aleman B.M.P., Noordijk E.M., Fermé C., Thomas J., Stamatoullas A., Fruchart C., Brice P., Gaillard I., Doorduijn J.K., Sebban C., Smit W.G.J.M., Bologna S., Roesink J.M., Ong F., André M.P.E., Raemaekers J.M.M., Henry-Amar M., Kluin-Nelemans H.C. | 2012 | Parenthood in Survivors of Hodgkin Lymphoma: An EORTC-GELA General Population Case-Control Study. | Journal of Clinical Oncology | 30 (31) | 3854-3863 | <https://doi.org/10.1200/JCO.2011.40.8906> | Receiving therapy in adulthood. |
| Lones M.A., Raphael M., McCarthy K., Wotherspoon A., Terrier-Lacombe M.-J., Ramsay A.D., MacLennan K., Cairo M.S., Gerrard M., Michon J., Patte C., Pinkerton. | 2012 | Primary Follicular Lymphoma of the Testis in Children and Adolescents. | Journal of Pediatric Hematology/Oncology | 34 (1) | 68-71 | <https://doi.org/10.1097/MPH.0b013e31820e4636> | No fertility outcomes. |
| Signorello L.B., Mulvihill J.J., Green D.M., Munro H.M., Stovall M., Weathers R.E., Mertens A.C., Whitton J.A., Robison L.L., Boice Jr. J.D. | 2012 | Congenital Anomalies in the Children of Cancer Survivors: A Report From the Childhood Cancer Survivor Study. | Journal of Clinical Oncology | 30 (3) | 239-245 | <https://doi.org/10.1200/JCO.2011.37.2938> | Non-relevant chemotherapy concentrated study. |
| Romerius P., Ståhl O., Moëll C., Relander T., Cavallin-Ståhl E., Wiebe T., Giwercman Y.L., Giwercman A. | 2011 | High risk of azoospermia in men treated for childhood cancer. | International Journal of Andrology | 34 (1) | 69-76 | <https://doi.org/10.1111/j.1365-2605.2010.01058.x> | Vincristine not part of chemotherapy protocols. |
| Cai JY, Tang JY, Pan C, Xu M, Xue HL, Zhou M, Dong L, Ye QD, Jiang H, Shen SH, Chen J. | 2010 | Results of RS-99 protocol for childhood solid tumors. | World Journal of Pediatrics | 6 (1) | 43-49 | <https://doi.org/10.1007/s12519-010-0005-6> | No fertility outcomes. |
| Green DM, Sklar CA, Boice JD Jr, Mulvihill JJ, Whitton JA, Stovall M, Yasui Y. | 2009 | Ovarian failure and reproductive outcomes after childhood cancer treatment: results from the Childhood Cancer Survivor Study. | Journal of Clinical Oncology | 27 (14) | 2374-2381 | <https://doi.org/10.1200/JCO.2008.21.1839> | Review. |
| Mitchell RT, Saunders PTK, Sharpe RM, Kelnar CJH, Wallace WHB. | 2009 | Male fertility and strategies for fertility preservation following childhood cancer treatment. | Endocrine Development | 15 | 101-134 | <https://doi.org/10.1159/000207612> | Review. |
| Frias S, Van Hummelen P, Meistrich ML, Lowe XR, Hagemeister FB, Shelby MD, Bishop JB, Wyrobek AJ. | 2003 | NOVP chemotherapy for Hodgkin's disease transiently induces sperm aneuploidies associated with the major clinical aneuploidy syndromes involving chromosomes X, Y, 18, and 21. | Cancer Research | 63 (1) | 44-51 | DOI unavailable | Diagnosis in adulthood. |
| Cicognani A, Pasini A, Pession A, Pirazzoli P, Burnelli R, Barbieri E, Mazzanti L, Cacciari E. | 2003 | Gonadal function and pubertal development after treatment of a childhood malignancy. | Pediatric Endocrinology and Metabolism | 16 Suppl 2 | 321-326 | DOI unavailable | Review. |
| Randi ML, Rossi C, Fabris F, Girolami A. | 2000 | Essential thrombocythemia in young adults: major thrombotic complications and complications during pregnancy--a follow-up study in 68 patients. | Clinical Applications of Thrombosis Hemostasis | 6 (1) | 31-35 | <https://doi.org/10.1177/107602960000600105> | Non-relevant chemotherapy concentrated study with effects on fertility. |
| Paulino AC, Wen BC, Brown CK, Tannous R, Mayr NA, Zhen WK, Weidner GJ, Hussey DH. | 2000 | Late effects in children treated with radiation therapy for Wilms' tumor. | International Journal of Radiation Oncology Biology, Physics | 46 (5) | 1239-1246 | <https://doi.org/10.1016/s0360-3016(99)00534-9> | No vincristine-concentrated fertility outcomes. |
| Sklar CA, LaQuaglia MP. | 2000 | The long-term complications of chemotherapy in childhood genitourinary tumors. | Urology Clinics of North America | 27 (3) | 563-568 | <https://doi.org/10.1016/s0094-0143(05)70103-8> | Review. |
| Cicognani A, Cacciari E, Pasini A, Burnelli R, De Iasio R, Pirazzoli P, Paolucci G. | 2000 | Low serum inhibin B levels as a marker of testicular damage after treatment for a childhood malignancy. | European Journal of Pediatrics | 159 (1-2) | 103-107 | <https://doi.org/10.1007/s004310050021> | Not clear-cut effects attributed to vincristine-contained regimens. |
| Göbel U, Calaminus G, Engert J, Kaatsch P, Gadner H, Bökkerink JP, Hass RJ, Waag K, Blohm ME, Dippert S, Teske C, Harms D. | 1998 | Teratomas in infancy and childhood. | Medical and Pediatric Oncology | 31 (1) | 8-15 | [https://doi.org/10.1002/(sici)1096-911x(199807)31:1<8::aid-mpo2>3.0.co;2-h](https://doi.org/10.1002/(sici)1096-911x(199807)31:1%3c8::aid-mpo2%3e3.0.co;2-h) | Non-relevant chemotherapy concentrated study with effects on fertility. |
| Li CK, Shing MM, Chik KW, Kwan WH, Lai DH, Leung TF, Yuen PM. | 1998 | Isolated testicular relapse after bone marrow transplant with total body irradiation and testicular boost in acute lymphoblastic leukemia. | Bone Marrow Transplant | 22 (4) | 397-399 | <https://doi.org/10.1038/sj.bmt.170134> | No fertility outcomes. |
| Schmiegelow ML, Sommer P, Carlsen E, Sønksen JO, Schmiegelow K, Müller JR. | 1998 | Penile vibratory stimulation and electroejaculation before anticancer therapy in two pubertal boys. | Journal of Pediatric Hematology and Oncology | 20 (5) | 429-430 | https://doi.org/10.1097/00043426-199809000-00004 | No fertility outcomes. |
| Robbins WA, Meistrich ML, Moore D, Hagemeister FB, Weier HU, Cassel MJ, Wilson G, Eskenazi B, Wyrobek AJ. | 1997 | Chemotherapy induces transient sex chromosomal and autosomal aneuploidy in human sperm. | Nature Genetics | 16 (1) | 74-78 | <https://doi.org/10.1038/ng0597-74> | Diagnosis in adulthood. |
| Blumenfeld Z, Haim N. | 1997 | Prevention of gonadal damage during cytotoxic therapy. | Annals of Medicine | 29 (3) | 199-206 | <https://doi.org/10.3109/07853899708999337> | Women-concentrated study. |
| Kulkarni SS, Sastry PS, Saikia TK, Parikh PM, Gopal R, Advani SH. | 1997 | Gonadal function following ABVD therapy for Hodgkin's disease. | Americal Journal of Clinical Oncology | 20 (4) | 354-357 | <https://doi.org/10.1097/00000421-199708000-00006> | Age at treatment not clearly illustrated. |
| Mackie EJ, Radford M, Shalet SM. | 1996 | Gonadal function following chemotherapy for childhood Hodgkin's disease. | Medical and Pediatric Oncology | 27 (2) | 74-78 | [https://doi.org/10.1002/(SICI)1096-911X(199608)27:2<74::AID-MPO2>3.0.CO;2-Q](https://doi.org/10.1002/(SICI)1096-911X(199608)27:2%3c74::AID-MPO2%3e3.0.CO;2-Q) | Vincristine not part of chemotherapy protocols. |
| Schellong G. | 1996 | Treatment of children and adolescents with Hodgkin's disease: the experience of the German-Austrian Paediatric Study Group. | Bailliere's Clinical Haematology | 9 (3) | 619-634 | <https://doi.org/10.1016/s0950-3536(96)80030-8> | Not specifying whether there are prepubertal patients. |
| Schellong G, Brämswig JH, Hörnig-Franz I, Schwarze EW, Pötter R, Wannenmacher M. | 1994 | Hodgkin's disease in children: combined modality treatment for stages IA, IB, and IIA. Results in 356 patients of the German/Austrian Pediatric Study Group. | Annals of Oncology | 5 Suppl 2 | 113-115 | <https://doi.org/10.1093/annonc/5.suppl_2.s113> | Not specifying whether there are prepubertal patients. Below 16 years of age. |
| Sahjpaul RL, Ramsay DA, de Veber LL, Del Maestro RF. | 1993 | Brain metastasis from clear cell sarcoma of the kidney--a case report and review of the literature. | Journal of Neuro-oncology | 16 (3) | 221-226 | <https://doi.org/10.1007/BF01057037> | Review. |
| David J, Vouyiouka O, Ansell BM, Hall A, Woo P. | 1993 | Amyloidosis in juvenile chronic arthritis: a morbidity and mortality study. | Clinical and Experimental Rheumatology. | 11 (1) | 85-90 | Gonadal function and pubertal development after treatment of a childhood malignancy. | No cancer reference; vincristine not part of protocols. |
| Nicholson HS, Byrne J. | 1993 | Fertility and pregnancy after treatment for cancer during childhood or adolescence. | Cancer | 71 (10 Suppl) | 3392-3399 | [https://doi.org/10.1002/1097-0142(19930515)71:10+<3392::aid-cncr2820711743>3.0.co;2-f](https://doi.org/10.1002/1097-0142(19930515)71:10+%3c3392::aid-cncr2820711743%3e3.0.co;2-f) | Review. |
| Schellong G, Brämswig JH, Hörnig-Franz I. | 1992 | Treatment of children with Hodgkin's disease--results of the German Pediatric Oncology Group. | Annals of Oncology | 3 Suppl 4 | 73-76 | <https://doi.org/10.1016/0022-3468(90)90381-I> | Not specifying whether there are prepubertal patients. Below 16 years of age. |
| Hawkins MM. | 1991 | Is there evidence of a therapy-related increase in germ cell mutation among childhood cancer survivors? | Journal of the National Cancer Institute | 83 (22) | 1643-1650 | <https://doi.org/10.1093/jnci/83.22.1643> | Prepubertal status at therapy not clear. |
| Levy MJ, Stillman RJ. | 1991 | Reproductive potential in survivors of childhood malignancy. | Pediatrician | 18 (1) | 61-70 | DOI unavailable | Review. |
| Huddart SN, Mann JR, Gornall P, Pearson D, Barrett A, Raafat F, Barnes JM, Wallendsus KR. | 1990 | The UK Children's Cancer Study Group: testicular malignant germ cell tumours 1979-1988. | Journal of Pediatric Surgery | 25 (4) | 406-410 | <https://doi.org/10.1016/0022-3468(90)90381-I> | No fertility outcomes. |
| Siimes MA, Rautonen J. | 1990 | Small testicles with impaired production of sperm in adult male survivors of childhood malignancies. | Cancer | 65 (6) | 1303-1306 | [https://doi.org/10.1002/1097-0142(19900315)65:6<1303::aid-cncr2820650608>3.0.co;2-d](https://doi.org/10.1002/1097-0142(19900315)65:6%3c1303::aid-cncr2820650608%3e3.0.co;2-d) | Non-relevant chemotherapy concentrated study. |
| Miller DR, Leikin SL, Albo VC, Palmer NF, Sather HN, Hammond GD. | 1990 | The prognostic value of testicular biopsy in childhood acute lymphoblastic leukemia: a report from the Childrens Cancer Study Group. | Journal of Clinical Oncology | 8 (1) | 57-66 | <https://doi.org/10.1200/JCO.1990.8.1.57> | No fertility outcomes. |
| Shebib S, Sabbah RS, Sackey K, Akhtar M, Aur RJ. | 1989 | Endodermal sinus (yolk sac) tumor in infants and children. A clinical and pathologic study: an 11 year review. | The American Journal of Pediatric Hematology/Oncology. | 11 (1) | 36-39 | <https://doi.org/10.1097/00043426-198921000-00009> | No fertility outcomes reported, for boys receiving vincristine. |
| Miller DR, Leikin SL, Albo VC, Sather H, Hammond GD. | 1989 | Three versus five years of maintenance therapy are equivalent in childhood acute lymphoblastic leukemia: a report from the Childrens Cancer Study Group. | Journal of Clinical Oncology | 7 (3) | 316-325 | <https://doi.org/10.1200/JCO.1989.7.3.316> | No fertility outcomes. |
| Raney RB Jr, Tefft M, Maurer HM, Ragab AH, Hays DM, Soule EH, Foulkes MA, Gehan EA. | 1988 | Disease patterns and survival rate in children with metastatic soft-tissue sarcoma. A report from the Intergroup Rhabdomyosarcoma Study (IRS)-I. | Cancer | 62 (7) | 1257-1266 | [https://doi.org/10.1002/1097-0142(19881001)62:7<1257::aid-cncr2820620703>3.0.co;2-k](https://doi.org/10.1002/1097-0142(19881001)62:7%3c1257::aid-cncr2820620703%3e3.0.co;2-k) | No fertility outcomes of chemotherapy regimens. |
| Laberge JM, Nguyen LT, Homsy YL, Doody DP. | 1987 | Bilateral Wilms' tumors: changing concepts in management. | Journal of Pediatric Surgery | 22 (8) | 730-735 | <https://doi.org/10.1016/s0022-3468(87)80615-2> | No fertility outcomes. |
| Kreuser E.D., Xiros N., Hetzel W.D., Heimpel H. | 1987 | Reproductive and endocrine gonadal capacity in patients treated with COPP chemotherapy for Hodgkin's disease. | Journal of Cancer Research and Clinical Oncology | 113 (3) | 260-266 | <https://doi.org/10.1007/BF00396383> | Not clear whether the therapy is administered during prepuberty. |
| Allen JC, Kim JH, Packer RJ. | 1987 | Neoadjuvant chemotherapy for newly diagnosed germ-cell tumors of the central nervous system. | Journal of Neurosurgery | 67 (1) | 65-70 | <https://doi.org/10.3171/jns.1987.67.1.0065> | Vincristine not part of chemotherapy protocols. |
| Byrne J, Mulvihill JJ, Myers MH, Connelly RR, Naughton MD, Krauss MR, Steinhorn SC, Hassinger DD, Austin DF, Bragg K, et al. | 1987 | Effects of Treatment on Fertility in Long-Term Survivors of Childhood or Adolescent Cancer. | New England Journal of Medicine | 317 (21) | 1315-1321 | <https://doi.org/10.1056/NEJM198711193172104> | Age not clearly illustrated for inclusion of childhood cancer survivors. |
| Allen JC, Bosl G, Walker R. | 1985 | Chemotherapy trials in recurrent primary intracranial germ cell tumors. | Journal of Neuro Oncology | 3 (2) | 147-152 | <https://doi.org/10.1007/BF02228891> | Vincristine not part of chemotherapy protocols. |
| [Giuliana Anelli](https://onlinelibrary.wiley.com/action/doSearch?ContribAuthorRaw=Anelli%2C+Giuliana), [Giusi Cozzi](https://onlinelibrary.wiley.com/action/doSearch?ContribAuthorRaw=Cozzi%2C+Giusi), [Emilia Franchi](https://onlinelibrary.wiley.com/action/doSearch?ContribAuthorRaw=Franchi%2C+Emilia), [Mariella Parlavecchia](https://onlinelibrary.wiley.com/action/doSearch?ContribAuthorRaw=Parlavecchia%2C+Mariella), [Franco Chiara](https://onlinelibrary.wiley.com/action/doSearch?ContribAuthorRaw=Chiara%2C+Franco), [Giuseppe Masera](https://onlinelibrary.wiley.com/action/doSearch?ContribAuthorRaw=Masera%2C+Giuseppe), [Marina Camatini](https://onlinelibrary.wiley.com/action/doSearch?ContribAuthorRaw=Camatini%2C+Marina) | 1984 | Responsiveness of testis morphology to chemotherapy in childhood leukemia | The Anatomical Record | 209(4) | 491-500 | https://doi.org/10.1002/ar.1092090409 | Fertility outcome was testis histology only |
| Anton Brogger, Randi Beck Nicolaysen, Randi Nygaard, Helga Waksvik,  Peter Johan Moe, Jorgen Cohn. | 1984 | Structural chromosome aberrations in lymphocytes from  children with one to five years of total remission after  chemotherapy of acute lymphoblastic leukaemia. | Pediatric Hematology and Oncology | 1 (1) | 39-46 | DOI unavailable | Study not concerned with fertility outcomes of chemotherapy received. |
| Cunningham J, Mauch P, Rosenthal DS, Canellos GP. | 1982 | Long-term complications of MOPP chemotherapy in patients with Hodgkin's disease. | Cancer Treatment Reports | 66 (4) | 1015-1022 | DOI unavailable | Treatment was not performed in prepuberty. |
| Brodeur GM, Howarth CB, Pratt CB, Caces J, Hustu HO. | 1981 | Malignant germ cell tumors in 57 children and adolescents. | Cancer | 48 (8) | 1890-1898 | [https://doi.org/10.1002/1097-0142(19811015)48:8<1890::aid-cncr2820480830>3.0.co;2-d](https://doi.org/10.1002/1097-0142(19811015)48:8%3c1890::aid-cncr2820480830%3e3.0.co;2-d) | No fertility outcomes. |
| Armata J, Borkowski W, Kaczor Z. | 1981 | Treatment failures and severe complications in children with Hodgkin's disease. | Folia Haematolica - Int Mag Klin Morphol Blutforsch. | 108 (3) | 406-411 | DOI unavailable | Not in English. |
| Lendon M, Hann IM, Palmer MK, Shalet SM, Jones PH. | 1978 | Testicular histology after combination chemotherapy in childhood for acute lymphoblastic leukaemia. | Lancet | 2 (8087) | 439-441 | <https://doi.org/10.1016/s0140-6736(78)91442-3> | No fertility outcomes but comments on tubular morphology. |
| Prioleau G, Wilson CB. | 1976 | Endodermal sinus tumor of the pineal region: case report. | Cancer | 38 (6) | 2489-2493 | [https://doi.org/10.1002/1097-0142(197612)38:6<2489::aid-cncr2820380638>3.0.co;2-j](https://doi.org/10.1002/1097-0142(197612)38:6%3c2489::aid-cncr2820380638%3e3.0.co;2-j) | No fertility outcomes. |

**Supplementary Table SIII** Detailed summary of full text publications included in the systematic review.

| **Study design** | **Participants** | **Treatment** | **Fertility outcomes** | **Control group** | **Additional Comments** |
| --- | --- | --- | --- | --- | --- |
| Kruseová, Jarmila et al., 2021. Semen analysis and treatment risk factors in long‐term survivors of childhood cancer. *Andrologia*, 53(1), pp. e13853-n/a. | | | | | |
| Single-centre retrospective cohort study | Childhood survivors: 143 (66 pre-pubertal and 77 post-pubertal patients at cancer diagnosis).  Age: Median age at cancer diagnosis 13.7 years (range, 0.1-19.1 years) and a median time  of follow-up of 11.6 years (range, 5.1–32.0 years).  Diagnoses:  Hodgkin's lymphoma (n=81), Ewing's sarcoma (n=13), acute lymphoblastic leukemia (n=10), non-Hodgkin's lymphoma (n=10), soft tissue sarcoma (n=9) or other solid tumours (n=20). | Chemotherapy:  Five chemotherapeutic groups were compared: antitumor antibiotics, alkylating agents,  topoisomerase and mitotic inhibitors, platinum-based agents and  antimetabolites.  Radiotherapy(n=34)  Abdominal irradiation with a median dose 24.8 Gy (range, 15–40 Gy) (n=26).  Cranial irradiation with a median dose 40.2 Gy (range, 12– 55.6 Gy) (n=8).  Cranial + spinal irradiation 25 Gy (n=3). | Semen quality (sperm density, progressive motility and spermia).  Definitions  *Azoospermia:* complete absence of spermatozoa  within the ejaculate. *Oligozoospermia:* low sperm density; the number of spermatozoa is ≤15 × 10^6^/ml.  *Normozoospermia:* normal sperm density; the number of spermatozoa  is ≥15 × 10^6^/ml. *Asthenozoospermia:* reduced sperm progressive motility of ˂32%.  Semen analysis:  65% of the survivors had abnormal semen quality i.e.:  Oligozoospermia (20%)  Azoospermia (37%)  Asthenozoospermia (n=11, 8%).  Normozoospermia (35%)  Survivors with azoospermia had higher spermia (semen volume) than the controls.  Hormone levels:  Survivors with an abnormal semen analysis have increased FSH and LH levels with time since diagnosis.  An increase in LH was also observed in the normal semen analysis. | Yes (200 males with no history of cancer) | Tanner criteria were used to assess the pubertal status at the time of cancer diagnosis.  Prepubertal status median age was 7.76 years (range, 0.12–12.56 years) and post-pubertal status was 15.24 years (range, 11.22–18.52 years).  No significant difference was found in terms of abnormal semen analysis between the two groups (p=0.09).  For assessing cancer-related risk factors and hormone levels, survivors who had azoospermia, oligozoospermia or asthenozoospermia were combined into one group.  Hormonal normal range was FSH= 1–8 IU/L and LH= 1.2–10 IU/L. Any result above the upper limit for FSH and LH were considered abnormal. |
| Utriainen, Pauliina et al., 2019. Gonadal Failure Is Common in Long-Term Survivors of Childhood High-Risk Neuroblastoma Treated with High-Dose Chemotherapy and Autologous Stem Cell Rescue. *Frontiers in endocrinology (Lausanne),* 10, p.555. | | | | | |
| Multi-centre cohort | Childhood survivors: n=20; 9 males long-term (>10 years) survivors of HR-NBL.  Age at diagnosis: 1.6 years (range, 0.2–3.6 years).  Age at examination:  Median age of 21.7 (range, 15.9–30.1).  Diagnosis:  High-risk Neuroblastoma (HR-NBL). | Induction chemotherapy:  COD, cyclophosphamide +vincristine+ DTIC; AAP, cisplatin+ doxorubicin.  Local control:  Removal of the original tumour in all (n=20) and local irradiation in 14/20.  Myeloablative therapy with autologous HSCT:  Total-body irradiation (TBI) in combination with chemotherapy in 10/20 (Eto+ carbo+ thiotepa/ melphalan/other chemotherapy combinations).  Retinoic acid:  All 20/20 patients and continued for varying time periods. | Gonadotrophins and Testicular volume:  When compared with control males, the survivors had smaller testicular size (8.5 vs. 39 ml) and lower inhibin B levels (<10 vs. 170ng/l).  Criteria of gonadal failure were fulfilled by 6/9 survivor males (i.e., absent puberty, small testicular size or increased FSH with need of androgen substitution).  One male had offspring, all treated without total-body irradiation and moderate dose of alkylating chemotherapy. | Yes  (Comprised of 20 Healthy age and sex-matched controls). | Gonadal failure: (A) absent development to puberty, (B) increased gonadotropins (FSH >10.4 IU/l in males) after spontaneous puberty in needing hormonal replacement therapy (HRT), (C) small testis size (<15 ml) post-pubertally as an indication of non-functional spermatogenesis.  Testis size exceeding 15 ml regarded as normal. |
| Beaud, Hermance et al., 2019. Sperm DNA integrity in adult survivors of paediatric leukemia and lymphoma: A pilot study on the impact of age and type of treatment. *PloS One*, 14(12), p.e0226262. | | | | | |
| Multi-centre cohort | Childhood survivors: 13 (n=6 before puberty, n=7 after puberty).  Age at diagnosis:  Pre-puberty (ages 9.2 ± 1.8, n=6) and post-puberty (ages 16.0 ± 0.4, n=7).  Diagnoses:  Acute lymphocytic leukemia (ALL), Hodgkin’s Lymphoma (HL) and Non-Hodgkin’s Lymphoma (NHL). | Chemotherapy:  Vinca Alkaloids (e.g., vincristine).  Alkylating agents (CED) (e.g., cyclophosphamide).  Anthracyclines (e.g., bleomycin).  *Individual cancer treatment illustrated as part of supplementary material of the paper.* | Semen analysis: DNA and chromatin abnormalities of sperm showed no difference when compared to the general population.  Individual values revealed that 5/13 CCS had sperm concentrations below the WHO standards of 15 million/ml; n=3 were azoospermic (10.9) and n=2 were oligozoospermic (1.4 million sperm/ml).  Hormone levels:  4 childhood cancer survivors who have FSH levels > 8IUs/L which is the clinical standard, suggest non-obstructive azoo/oligozoospermia. | Yes  (Healthy age and sex-matched controls with no history of cancer). | One individual in the control group, had a low percentage of total sperm motility and displayed abnormal sperm parameters according to the World Health Organization (WHO) standards.  Sperm from adult cancer patients could show that cancer and its treatment can induce sperm aneuploidy, chromatin damage, and epigenetic changes that persist years post-chemotherapy.  Exclusion criteria in the study were: 1) inability to provide informed consent; 2) receiving total body or pelvic radiation treatment. |
| Green, Daniel M et al., 2014. The cyclophosphamide equivalent dose as an approach for quantifying alkylating agent exposure: A report from the childhood cancer survivor study. *Pediatric Blood & Cancer*, 61(1), pp.53–67. | | | | | |
| Multi-centre cohort | Survivors: 4,579.  CCSS identified and recruited all survivors meeting eligibility criteria at 26 institutions in the United States (n=25) and Canada (n=1).  Age at diagnosis:  5-20  Diagnoses: Leukemia, lymphoma, central nervous system cancer, neuroblastoma, bone or soft tissue sarcoma, or kidney cancer. | Chemotherapy:  Various agents used to estimate the alkylating agent dose equivalent. | In the multivariable models for both CED and AAD, the hazard ratio (HR) ***for siring a pregnancy*** is associated significantly with alkylating agent exposure in a dose-dependent manner.  HR for the effect of vincristine when administered with CED-based regimens was 1.04 with a 95% CI of 0.85-1.28 and P value= 0.690. Whilst for AAD HR was 1.07 with a 95% CI of 0.87-1.32 and P value= 0.53.  The HR for both CED and AAD-based regimens, translate that exposure of patients to vincristine does not have a clear-cut association and an increased risk for being responsible of not siring a pregnancy. The P values also suggest that there is no significant difference when vincristine is given in terms of the number of pregnancies that are going to result after chemotherapy administration. | No | The CCSS represents the largest and most extensively characterized cohort of childhood and adolescent cancer survivors in North America. It serves as a resource for addressing important issues such as endocrine and reproductive outcomes.  This project was undertaken to develop a simple method for the normalization of alkylating agent exposure to units of a single drug.  Comparison of the performance of the Cyclophosphamide Equivalent Dose (CED), a unit for quantifying alkylating agent exposure independent of study population, to the AAD. |
| Shiraishi, Koji & Matsuyama, Hideyasu, 2014. Microdissection Testicular Sperm Extraction and Salvage Hormonal Treatment in Patients With Postchemotherapy Azoospermia. *Urology (Ridgewood, N.J.),* 83(1), pp.100–106. | | | | | |
| Single-centre cohort | Survivors: 26 patients with post chemotherapy azoospermia (PCA).  Μean age: 34.6 years (range, 23-42).  Mean interval from chemotherapy to micro-TESE: 14.8 years (range, 7-25).  Age at diagnosis: Not specified.  However, age at chemotherapy end for the group we are concerned with, was 9.3 years.  Diagnoses:  Τesticular cancer (8 patients, 31%), Hodgkin’s lymphoma, non-Hodgkin’s lymphoma, leukemia, neuroblastoma, osteosarcoma, and malignant pheochromocytoma. | Various Chemotherapy treatments, but for prepubertal ALL (n=4): Cyclophosphamide (CPA), Vincristine (VCR), Daunomycin (DNR). | Gonadotrophins and testicular volume:  The 4 patients (treated for ALL) at a chemotherapy end of 9.3 years of age. Duration to infertility treatment had been 24.2 years, with hormonal levels of LH: 7.4 miU/L; FSH: 20.1 miU/L; Testosterone: 407.0 ng/dL; testis volume 10.7 mL; testicular pathology of ‘Sertoli-cell only’ in all 4 of the patients; and sperm retrieval achieved in only ¼ patients (25%).  ***All cohort results:***  Pregnancy and fertility outcomes:  Overall, 7 pregnancies had been achieved (27%), with 5 resulting in live birth delivery (19%). All new-borns were described as healthy, with no observation of birth defects.  The average testicular volume was 12.6 mL.  Gonadotrophins:  Mean LH, FSH, and testosterone levels of 11.7, 23.6 mIU/L, and 349.3 ng/dL, respectively, thus indicating primary spermatogenic failure.  Testicular histologic diagnoses: Hypospermatogenesis in 5 patients (19%), maturation arrest in 5 patients (19%), and SCO in 16 patients (62%). | No | Patients with PCA after testicular cancer treatment could retrieve the highest rate of sperm (75%), whilst exposure to alkylating agent regimens resulted in lower retrieval rates. Thus, patients with positive sperm results had a significantly higher prevalence of testicular cancer (P<0.05), and the frequency of alkylating agents had been lower (P<0.05) when compared with the sperm-negative group.  Clinical pregnancy was concluded via the presence of a fetal heartbeat on a transvaginal ultrasound. |
| Hamre, Hanne et al., 2012. Gonadal function and parenthood 20 years after treatment for childhood lymphoma: A cross‐sectional study. *Pediatric Blood & Cancer*, 59(2), pp.271–277. | | | | | |
| Cross-sectional study | Survivors: 74 male participants (n=20 prepubertal)  Age at diagnosis: median 13.3 years (range, 3.0–17.8 years).  At survey: 33.6 years (range, 19.0–54.5 years).  Diagnoses:  Hodgkin’s Lymphoma (HL) or non-Hodgkin lymphoma (NHL). | Heterogeneous treatment strategies extracted from medical records and sub-divided into low, medium, high toxicities. | Hypogonadism was observed in 7 of 66 males (11%).  Semen analysis:  Azoospermia was seen in 12 of 42 childhood lymphoma survivors; 7 were oligospermic and 23 had normospermia.  The proportion of azoospermic men and time to pregnancy increased with treatment burden (P=0.009, P=0.045).  *Age-adjusted limits for hormones*: FSH ≤12 U/L, LH ≤10 U/L, testosterone 10.0–31.0 nmol/L. Thus, male hypogonadism was defined as LH level ≥10.0 U/L or testosterone <10 nmol/L.  Gonadotrophins:  Median testosterone:  18.4 nmol/L (range: 8.9–35.8); median LH: 5.5 U/L (range: 2.0–12.3); median FSH: 6.2 U/L (range: 1.4–33.9).  Increasing treatment toxicity, significantly increases the levels of LH within normal range (P=0.025).  Twenty males (31%) had FSH levels above age-adjusted limits. Also, FSH significantly increased with treatment burden (P=0.002).  Pregnancy outcomes:  Exploration of any pregnancy, involuntary infertility, use of assisted fertilization, and testosterone substitution.  21/33 (64%) male had achieved post-treatment parenthood (P<0.001). Median age at first pregnancy 25.8 years (P=0.039). | No | Questionnaire, clinical examination, and blood/semen analysis.  Hypogonadism was determined by levels of gonadal hormones based on LH, FSH, testosterone.  Patients receiving treatment for a second malignancy were excluded. |
| Zaletel, Lorna Zadravec, Bratanic, Nevenka & Jereb, Berta, 2010. Gonadal function in patients treated for Hodgkin′s disease in childhood. *Radiology and Oncology*, 44(3), pp.187–193. | | | | | |
| Single-centre cohort | Survivors: 40 males.  Age at diagnosis:  median age of 13 (range, 3-16 years).  Diagnosis:  Hodgkin’s disease (HD). | Chemotherapy:  Out of the 24 males with primary hypogonadism (PH), 22 received combination chemotherapy (≥ 6 cycles of alkylating agent and procarbazine consisting ChT) and RT (pelvic irradiation in 8 of them), whilst 2 had pelvic RT only. Patients who had LC damage/dysfunction received slightly higher doses of P (median 7.4 g/m^2^) than those with normal function (median 6.5 g/m^2^).  Protocols: MOPP, MOPP-ABV, MOPP/ABVD, LOPP, COPP(A) and OPPA.  Radiation:  n=19 with RT above the diaphragm with 20-40 (median 30) Gy; n=8 with RT to the upper abdomen with 24-49 (median 30) Gy; n=11 RT to the pelvis with 22-45 (median 30) Gy. | Endocrine dysfunction:  PH was seen in 24/40 (60%) of male patients with evidence of damage of the germinal epithelium (20/35 i.e., 57% who had a primary treatment and in 4/5 males i.e., 80% treated for relapse), 4 patients were found to have a potential damage of Leydig cells (LC) (low level of testosterone in one patient), and 10 had also evidence of their dysfunction.  Among the 7 males who received 6 cycles of MOPP/ABVD but non-pelvic RT, only one gave normal endocrine tests. Along the same lines, 10 patients receiving 6 cycles of LOPP with non-pelvic RT, only 4 had gonadal function which was normal. Normal testicular gonadal function was seen in 3 patients who received 1 or 2 cycles of MOPP or OPPA.  Semen Analysis:  6/24 patients with PH (25%) were azoospermic.  5/24 fathered a child. | No |  |
| Daniel M. Green et al., 2010. Fertility of Male Survivors of Childhood Cancer: A Report From the Childhood Cancer Survivor Study. *Journal of Clinical Oncology*, 28(2), pp.332–339. | | | | | |
| Multicentre cohort of the Childhood Cancer Survivor Study | Survivors: 6,224 aged 15 to 44 years (and not surgically sterile).  Age at diagnosis: 0-4; 5-9; 10-14.  Diagnoses:  Multiple (Leukemia, CNS, Hodgkin’s lymphoma, Non-Hodgkin’s lymphoma, Kidney (Wilms disease), Neuroblastoma, Soft tissue sarcoma, Bone Cancer). | Summed alkylating agent scores and Individual Chemotherapy agents analysed, along with irradiation as part of the regimen. | Pregnancy and fertility outcomes:  The hazard ratio (HR) for a participant ever siring a pregnancy was 0.56 (95% CI, -0.49-0.63; P<0.001) compared with their siblings.  The HR for siring a pregnancy, adjusted for marital status, race/ethnicity, and educational attainment, for CCSS participants who had an AAD score= 0, a hypothalamic/pituitary or testes radiation dose of 0 Gy, was 0.91 (95% CI, 0.73 to 1.14; P=0.41).  Participants who received testicular radiation at a dose ≤7.5 Gy were as likely to sire a pregnancy when compared with patients not receiving radiation to the testis (HR, 1.62; 95% CI, 0.39-6.71; P=0.51).  HR for siring a pregnancy was inversely proportional to the summed AAD score (P≤0.001 (linear trend)). | Yes (Healthy Siblings) | Reviewed the fertility of male Childhood Cancer Survivor Study survivor and sibling cohorts  who completed a questionnaire  Risk factors for siring a pregnancy were evaluated using Cox proportional hazards models.  Pregnancies resulting from assisted reproductive technology were excluded from the analysis. |
| Nurmio, Mirja et al., 2009. Effect of Childhood Acute Lymphoblastic Leukemia Therapy on Spermatogonia Populations and Future Fertility. *The Journal of Clinical Endocrinology and Metabolism,* 94(6), pp.2119–2122. | | | | | |
| Multicentre  study | Survivors: 23 prepubertal boys (including 28 testicular biopsies).  Age at diagnosis: Mean age of 5.7±2.9 years.  Diagnosis: Acute Lymphoblastic Leukemia (ALL). | Treatment for leukemia with multiple chemotherapy regimens, with the total dose differing according to the type of therapy received.  n=4 as part of the high-risk group received cerebral irradiation (24 Gy), but not spinal irradiation.  Testicular relapse patients received a multidrug chemotherapy regimen, along with testicular and cranial irradiation of the same dose. | Testicular function when considering levels of inhibin B, FSH, testosterone, LH, testicular volume, and sperm count, was monitored at 17.0 ± 1.9 years after the initial diagnosis.  Testicular size: mean ± SEM: 19.3 ± 1.7 ml.  Gonadotrophins:  Levels of FSH (3.2 ± 0.5 IU/L), LH (4.2 ± 0.9 IU/L), testosterone (19 ± 3 nmol/L), and inhibin-B (225 ± 38 Ng/L); these values were comparable to healthy Finnish young men.  Semen samples:  n=3 providing semen samples had normal mean sperm counts of 61 ± 3 x 10^6^/ml, with two of them fathering a child. | No |  |
| Krawczuk-Rybak, Maryna et al., 2009. TESTICULAR FUNCTION AFTER TREATMENT FOR ACUTE LYMPHOBLASTIC LEUKEMIA (ALL) IN PREPUBERTAL AND PUBERTAL BOYS. *Pediatric Hematology and Oncology*, 26(7), pp.504–514. | | | | | |
| Single-centre cohort | Survivors: 59 males (51 prepubertal and 8 pubertal).  Age:  Group 1 (n=27): mean age of 8.4 ± 2.2 years, in Tanner stage I or II. They were treated 1.9 ± 1.3 years earlier for ALL (mean age at the diagnosis 4.3 ± 1.7 years).  Group 2 (n=32) (Tanner III–V): mean age of 15.9 ± 2.6, diagnosed 5.3 ± 3.5 years earlier, at the mean age of 7.9 ± 4.3.  Treatment at prepubertal stage:  Mean age 6.1 ± 2.7.  Diagnosis:  Acute lymphoblastic leukemia (ALL). | Radiotherapy:  Group 1: n=8, CNS (all those treated according to NY protocol received 18 Gy and 3 according to BFM protocol received 12 Gy).  Group 2 (n=18): n=6 patients received 18 Gy (2 NY protocol and 4 BFM protocol) and n=12 (BFM) received 12 Gy.  Chemotherapy (g/m^2^):  Prednisone (4.89–8.25 BFM, 20.16 NY)  Daunorubicine (0.12–0.24 BFM, 0.5 NY)  Cyclophosphamide (3.0 BFM, 17.4 NY)  6-Mercaptopurine (38.2–48.8 BFM, 16.8 NY)  6-Thioguanine (0.84 BFM, 3.54 NY)  Cytarabine (1.8 BFM, 4.56 NY)  Vincristine (0.012–0.03 BFM, 0.475 NY)  Methotrexate iv (20.0 BFM, 2.2–3.25 NY)  l-Asparaginase iv (12 × 10.000 BFM, 18 × 25.000 NY)  Methotrexate p.o. (2.66–3.65 BFM)  Dexamethasone (0.21 BFM)  Methotrexate iv (20.0 BFM, 2.2–3.25 NY)  l-Asparaginase iv (12 × 10.000 BFM, 18 × 25.000 NY)  Methotrexate p.o. (2.66–3.65 BFM)  Dexamethasone (0.21 BFM) | Serum inhibin B, FSH, testosterone, LH, and testicular volume were measured. Both groups illustrated lower mean values of inhibin B when compared to the control, whereas the other analyzed parameters were comparable. The inhibin B: FSH ratio was reduced as compared to the control. Testicular volume was lower.  Hormone levels:  Group 1: Inhibin B:FSH ratio did not differ from controls (54.3 ± 97.0 versus 104.8 ± 60.58, P=0.21).  Group 2: The mean value of inhibin B was lower (74.7 ± 71.2 ng/L versus 168.1 ± 77.2 ng/L, P<0.0001) than controls, and the mean testicular volume was lower (14.8 ± 5.1 versus 18.5 ± 4.7 mL, P=0.01) when compared to healthy boys. | Yes (Healthy age and sex-matched controls) | Control group comprised of 21 prepubertal (mean age 7.87 ± 2.59; P=0.42; compared to age of group 1, Tanner I, II) and 20 pubertal (mean age 15.9 ± 2.8 p = 1; comparable to group 2, Tanner III–V) healthy participants.  Treatment for ALL has damaging effects on spermatogenesis, regardless of the type of therapy or the age at treatment. |
| Khadijeh A. Rafsanjani, Mohammad Faranoush, Amir A. Hedayatiasl, Parvaneh Vossough, 2007. Gonadal function and fertility in male survivors treated for Hodgkin’s disease in Iran. *Saudi Medical Journal,* 28 (11), pp. 1690-1693 | | | | | |
| Single-centre cohort | Survivors: 33.  Age at diagnosis:  9.1 years (range, 5-15 years).  Diagnosis:  Hodgkin’s disease (HD). | Chemotherapy:  n=29 (87.8%) received 6-8 cycles of MOPP/ABVD, from which 5 (15.1%) relapsed and received other protocols for therapy. n=3 received MOPP/ABVD + RT; a single patient received 10 cycles of MOPP alone. | Semen Analysis:  27/33 patients (81.8%) were azoospermic, 2 had severe oligospermia, 3 were oligospermic and one had a normal sperm count (58,000,000).  Secondary sexual characteristics have been normal for all.  Gonadotrophins:  FSH and LH levels in 6/33 were higher than normal; testosterone levels in 3/33 were below normal levels. The median level of FSH was 8 mIU/ml (range, 1-32); for LH was 5 mIU/ml (range, 0.1-14); for testosterone was 4.10 ng/ml (range, 0.1-14.10). Although low levels of testosterone were found in these 3 patients, they also consisted of normal levels of LH.  Testicular volume: 17.5 ml (range of 14-20 ml). | No | Severe oligospermia and higher FSH levels had a strong correlation (r=1). |
| Garolla, Andrea et al., 2006. Progress in the development of childhood cancer therapy. *Reproductive Toxicology (Elmsford, N.Y.)*, 22(2), pp.126–132. | | | | | |
| Single-centre cohort | Survivors: 33 young males.  Age at diagnosis:  range, 3-17 years.  Diagnosis:  Soft tissue or bone sarcomas. | Chemotherapy  Protocol RMS 79 (n=8): (VAC and CAV cycles) consisted in the administration of cyclophosphamide associated to vincristine and actinomycin-D.  Protocol RMS 88 (n=18): (VAIA III and IVA II cycles) consisted in the administration of ifosfamide associated to vincristine, actinomycin-D and Adriamycin.  Protocol RMS 96 (n=5): Constituted  by the therapy schemes of RMS 88 (VAIA III and IVA II) with the addition  of CEVAIE cycles (repeated three times). CEVAIE cycle consisted in three different chemotherapy schemes named A, B and C. Scheme A (CEV), constituted by the association of vincristine, carboplatin and epirubicin; Scheme B (IVA), constituted by  the administration of ifosfamide associated to vincristine and actinomycin-d;  Scheme C (IVE), constituted by the association of ifosfamide, vincristine and  etoposide.  Protocol ISG/SSGI (n=2):  Consisted in the administration of high doses metotrexate, cisplatin, adriamicin followed by ifosfamide.  *Group B:*  n=10 (40%) had received a higher dose than 60 g/m^2^ & n= 15 (60%) a dose lower than 60 g/m^2^. | Semen analysis:  A significant reduction of *testicular mean volume* in patients of group A (administered cyclophosphamide-based regimens) respect to patients of group B (administered ifosfamide-based regimens) (P<0.001).  Reduction of mean *sperm count* in subjects of group A, in terms of sperm concentration and total sperm count.  Aneuploidy:  Group B showed independently from therapy protocol higher mean percentage of sperm aneuploidies (1.8%).  Hormone levels:  FSH (23.1 ± 15.6 in group A versus 8.8 ± 10.2 in group B, P<0.05) and inhibin B plasma levels (51.8 ± 72.5 in group A versus 150.3 ± 139.5 in group B, P<0.05). Higher LH and lower testosterone concentrations for Group A. | Yes (Normozoospermic control subjects) | All patients who received chemotherapy pre- or post-puberty, had been administered the RMS 79, RMS 88, RMS 96 or ISG/SSGI protocols.  33 patients were subdivided in two groups: group A (8 subjects), patients who had received chemotherapy protocol that included cyclophosphamide and group B (25 subjects), patients who had received ifosfamide as an alkylating drug.  At chemotherapy treatment all subjects of group A were prepubertal. Conversely, among patients of group B, thirteen were prepubertal and the remaining (12 subjects) were post-pubertal. |
| Hobbie, Wendy L et al., 2005. Fertility in males treated for Hodgkins disease with COPP/ABV hybrid. *Pediatric Blood & Cancer*, 44(2), pp.193–196. | | | | | |
| Single-centre cohort | Survivors: 11 males (3 pre-pubertal at the time of treatment).  Age at diagnosis:  13 years (range: 6– 19 years).  Median time off therapy for these 11 patients was 6.5 years (range: 1.5–21 years).  Diagnosis:  Hodgkin’s disease (HD). | Chemotherapy  COPP/ABV hybrid:  Cyclophosphamide (2.4–3.6g/m^2^ ), vincristine, procarbazine, and prednisone (COPP) together with adriamycin, bleomycin, and vinblastine (ABV). | Testicular size was within normal limits for all study participants.  Semen analysis:  9/11 categorized as infertile; 7 of 9 were azoospermic.  Gonadotrophins:  5/9 infertile males had normal levels of FSH and LH and no association was found between fertility and gonadotropin status (P=0.49). All had normal testosterone levels (10/11 available). | No | Patients with pelvic irradiation or the ones who had undergone stem cell transplantation were excluded from the study.  2/3 pre-pubertal males were azoospermic. The only fertile pre-pubertal male received the 0.4 g/m^2^ of procarbazine.  WHO says that a normal sperm count consists of 20 million sperm per ejaculate, with 50% motility and 60% normal morphology.  However, fertility status and more precisely the definition of infertility was <5 million in this study. |
| Alebouyeh, M et al., 2005. Successful ambulatory treatment of Hodgkin's disease in Iranian children based on German-Austrian DAL-HD 85-90: Single institutional results. *Annals of Oncology*, 16(12), pp.1936–1940. | | | | | |
| Single-centre cohort | Survivors: n=40 (25 males).  Age at diagnosis:  Mean 8.8 years (range, 4-14 years).  Diagnosis:  Hodgkin’s Disease (HD). | Chemotherapy and Radiotherapy:  As part of the regimen, they all received cyclophosphamide or procarbazine.  Stage IA and IIA patients (n=15): either 2x OPA (n=6) (vincristine, prednisolone and doxorubicin), or 2x OPPA (n=4) (vincristine, prednisolone, procarbazin and doxorubicin) or OPEA (n=2) (vincristine, prednisolone, etoposide and doxorubicin); n=3 stage IIA patients with slow response, additionally received 4x CO(P)P (cyclophosphamide, vicristine, prednisolone in alternate courses and procarbazin).  Stages IIB, IIIA/B and IV: 2x OPPA, followed by 4x CO(P)P; n=29 (72.5%) received reduced radiotherapy (20–25 Gy); n=4 (stage I) to the involved field; n=25 (stage II and also III/IV with residual or mediastinal mass) to the upper mantel and n=3 additionally to spleen. | Semen parameters  n=3 demonstrated azoospermia upon routine follow-up; one of these achieved a second malignancy.  ↓ of procarbazine could compromise the outcome of the treatment (even if reducing testicular damage). | No |  |
| Kenney, Lisa B et al., 2001. High risk of infertility and long term gonadal damage in males treated with high dose cyclophosphamide for sarcoma during childhood. *Cancer*, 91(3), pp.613–621. | | | | | |
| Single-centre cohort | Survivors: 17 (10 characterised prepubertal at the initiation of treatment).  Age at diagnosis: Median=12 years (range, 4-19 years).  Age at follow-up: 25 years (range, 16-34 years).  Diagnosis of Childhood sarcomas:  Paratesticular rhadomyosarcoma (n=5), head/neck rhadomyosarcoma (n=4), Ewing’s sarcoma (n=4), other sarcomas including mucoepidermoid sarcoma, mesenchymal sarcoma, epithelial sarcoma, and undifferentiated sarcoma (n=4). | Chemotherapy:  High-dose cyclophosphamide therapy as part of a VAC (vincristine, actinomycin, and cyclophosphamide) or Adria-VAC (doxorubicin, vincristine, actinomycin, and cyclophosphamide) chemotherapy regimen.  ** All patients received vincristine, actinomycin D, and cyclophosphamide, and eight patients also received doxorubicin.  Radiation:  n=6 received radiation as part of their initial planned therapy (6 to the head/neck, 3 to an extremity, 1 to the chest, and 1 to the lumbar spine).  Genitourinary (GU) surgery:  Performed in 6 patients; n=5 underwent unilateral orchiectomy for paratesticular rhabdomyosarcoma and 1, unilateral orchiectomy for an undescended testicle. | Semen analysis:  Azoospermia, n=10 (58.8%)  Oligospermia, n=5 (29.4%)  Normal sperm count, n=2 (11.8%) receiving the lowest doses of cyclophosphamide (7.5 g/m^2^).  Gonadotrophins:  Baseline FSH level was elevated in 10/14 patients.  Testosterone levels were normal in 15/16 patients (93.8%).  Baseline LH level was elevated in 6/15 patients.  GnRH-stimulated LH  levels were 3 times that of baseline in 13/14 patients (92.9%), suggesting a  degree of Leydig cell insufficiency. | No | n=7 patients were pubertal or post pubertal. |
| Arush, Myriam Weyl Ben et al., 2000. MALE GONADAL FUNCTION IN SURVIVORS OF CHILDHOOD HODGKIN AND NON-HODGKIN LYMPHOMA. *Pediatric Hematology and Oncology*, 17(3), pp.239–245. | | | | | |
| Single-centre cohort | Survivors: 20 survivors of childhood  Hodgkin and non-Hodgkin lymphoma (9 prepubertal).  Age at diagnosis: 2.1-16.4 years.  Follow-up assessment: 4-19 years (median 9.8 years).  Diagnoses:  Ewing sarcoma (n=2), Rhabdomyosarcoma (n=1), Osteosarcoma (n=5), Synovial sarcoma (n=1), Hodgkin’s disease (n=13), Neuroblastoma (n=6), Medulloblastoma (n=3), Acute Lymphoblastic Leukaemia (n=2). | Inverted Y Radiotherapy (2320 cGy (1550±4000)): n=5  Chemotherapy: n=20  MOPP/ABVD protocol, n=9; COMP protocol, n=5; MOPP protocol, n=3; other protocols, n=3. | Definitions:  Normospermia = sperm count >20x 10^6^ spermatozoa per milliliter  (sp/mL)  Oligospermia = sperm count of 1x 10^5^-20x 10^6^ sp/mL  Severe oligospermia = sperm count of 1±100,000 sp/mL  Azoospermia = sperm count of 0 sp/mL  Semen analysis:  Normal values, 4/20 patients; oligospermia, 8/20 patients; azoospermia, 8/20 patients; 4/5 who received Inverted Y irradiation were azoospermic and 1 was severely oligospermic.  Hormone levels  FSH above normal level, 10/20 patients.  Normal values for testosterone and FSH were 3±9 ng/mL and 1±14 mIU/mL, respectively. | No | n=12 with Hodgkin disease were treated with MOPP  or MOPP/ABVD combination chemotherapy.  n=8 with non-Hodgkin lymphoma were treated with one of the following: COM  (cyclophosphamide, vincristine, methotrexate); COMP (cyclophosphamide, vincristine, methotrexate, prednisone); LSA_2_L_2_ (cyclophosphamide, vincristine, doxorubicin, asparaginase, thioguanine, methotrexate, 6-mercaptopurine); or NCI protocol (methotrexate, cyclophosphamide, doxorubicin, prednisone).  During irradiation treatment the testes were lead shielded.  n=9 were prepubertal (Tanner I), 3 were intrapubertal (Tanner II+III), and 8 were postpubertal (Tanner V) at the time of diagnosis. |
| Heikens, Janneke et al., 1996. Irreversible gonadal damage in male survivors of pediatric Hodgkin's disease. *Cancer*, 78(9), pp.2020–2024. | | | | | |
| Single-centre cohort | Survivors: 19 males (15 prepubescent).  Age at diagnosis: 11 years (range, 5-15 years).  Follow-up: Complete remission for 6 to 14 years (median of 10 years) after treatment (median 19 years (range, 16-27 years)).  Further follow-up examination at 14 years (range, 13-20 years).  Diagnosis: Hodgkin’s Disease | Chemotherapy:  MOPP (mechlorethamine, vincristine, procarbazine, and prednisone) before (n=15) or during puberty (n=4).  Radiation  Adjuvant radiotherapy treatment was given in n=8; n=6 received irradiation above the diaphragm, and n=2 were irradiated below the diaphragm (20 Gy on the para-aortal and splenic regions and 25 Gy on the inguinal region). | *Normospermia:* sperm concentration >20 million/ml, moderate *oligospermia* as >1-20 million/ml, and severe oligospermia as 51 million/ml. A patient was considered *azoospermic* when there were no spermatozoa.  Semen analysis:  Azoospermia in n=12; oligospermia in n=6; no recovery of spermatogenesis was seen at follow-up.  Testicular size was deemed small in all but one patient (mean, 11 ml; range, 5-15 ml).  No delayed onset of puberty observed.  FSH levels were elevated (mean, 14.4 ± 7.8 U/l) and increased over time (mean, 21.1 ± 10.5 U/l, P<0.001).  In 7 patients, LH was elevated, indicating Leydig cell dysfunction; also, in 4 of those patients, plasma testosterone was decreased.  In three other patients, the response of LH to GnRH was exaggerated with a normal basal LH and testosterone. | No | Testicular function between prepubescent and pubescent state at the time of treatment, illustrates an improved outcome in the younger patients. |
| Müller, H.L et al., 1996. Gonadal function of young adults after therapy of malignancies during childhood or adolescence. *European Journal of Pediatrics,* 155(9), pp.763–769. | | | | | |
| Single-centre cohort | Survivors: 33 patients.  Age at diagnosis:  14 years (range 3-17 years).  13 Tanner Stage 1  Diagnosis:  Hodgkin’s Disease (HD). | 29/33 (87%) male survivors received chemotherapy.  Chemotherapy:  10/33 (30%) male survivors received procarbazine, 24/33 (73%) male patients received alkylating drugs.  Radiation | Gonadotrophins:  Male participants had elevated serum levels for basal and GnRH-stimulated FSH and LH.  No differences were found between survivors and normal controls for serum concentrations of testosterone.  Survivors had lower testicular volumes when compared with controls. Testicular morphology differences were not detectable.  Semen analysis:  14/33 (42%) participants and 8/12 (73%) male controls were evaluated by spermiogram. Even if all normal controls were normospermic, only 2/14 long-term survivors exhibited normozoospermia (P<0.001); 3/14 survivors had oligozoospermia (SCD: 16, 18 and 19 x 10^6^/ml) and 9/14 (64%) were azoospermic. | Yes (Healthy age and sex-matched) | A positive correlation (r=0.74, P<0.001) was illustrated between testicular volume and sperm cell density (SCD) and a negative correlation (r=-0.69, P<0.001) between basal FSH and SCD.  Azoospermic survivors were treated with alkylating agents more often and received higher (P<0.05) gonadal doses of radiation when compared with normospermic childhood survivors.  All male survivors evaluated with basal FSH levels above the normal range (>10 IU/l) and testicular volumes below the normal range (<13 ml) were illustrative of azoospermia. However, survivors and normal controls who had normal basal FSH and testicular volumes were normospermic. |
| Dhabhar, Boman N et al., 1993. Gonadal function in prepubertal boys following treatment for hodgkin’s disease. *The American Journal of Pediatric Hematology/oncology,* 15(3), pp.306–310. | | | | | |
| Single-centre cohort | Survivors: 26 adults (<12 years for n=13)  Age at diagnosis: 12 (range, 4-15) years.  Age at follow-up:  17 (range, 15-23) years.  Median duration after termination of chemotherapy was 72 months.  Diagnosis:  Hodgkin’s disease (HD). | Chemotherapies used in combination: COPP (cyclophosphamide, 600 mg/m^2^; vincristine, procarbazine, and prednisolone).  MOPP (mechlorethamine, 6 mg/m^2^, on days 1 and 8; vincristine, 1.4 mg/m^2^ on days 1 and 8; procarbazine 100 mg/m^2^, and prednisolone, 40, for 14 mg/m^2^ days in all the cycles).  ABVD (Adriamycin, Bleomycin, Vinblastine, Dacarbazine) | Semen analysis (n=18): All azoospermic.  Hormonal analysis showed elevated mean levels of FSH and inhibin, whereas LH was only marginally elevated.  Normal values: FSH, 100-500 ng/ml; LH 40-200 ng/ml; and inhibin, <30 ng/ml. | Yes (Healthy age and sex-matched controls). | No evidence of sexual maturation at the time of therapy.  Exclusion of patients receiving infra-diaphragmatic radiation.  Gonadal functions were evaluated after attainment of secondary sexual characteristics.  Serum testosterone was not evaluated due to wide diurnal variations. |
| Sy Ortin, T.T et al., 1990. Gonadal status and reproductive function following treatment for Hodgkin's disease in childhood: The Stanford Experience. *International Journal of Radiation Oncology, Biology, Physics*, 19(4), pp.873–880. | | | | | |
| Single-centre cohort | Survivors: 148 young male patients (5 prepubescent with data on gonadal function).  Age at diagnosis: 2- 15 years with a median of 13 years.  Follow-up: 9 years, while the maximum follow-up was 26 years.  Diagnosis: Osteosarcoma | Chemotherapy:  MOPP chemotherapy (mechlorethamine, vincristine, procarbazine, and prednisone).  Among n=8 treated with radiation only, n=4 were able to father a child (n=3 after 40-45 Gy pelvic radiation, n=1 without pelvic radiation). n=3 others who received 30-44 Gy pelvic radiation were oligospermic when tested post-treatment. | Semen analysis in 10/12 (83%) treated with 6 cycles of MOPP with or without pelvic radiation revealed azoospermia with no evidence of recovery after a follow-up of 11 years.  Following prolonged azoospermia, 2/12 boys (17%) recovered fertility, with normalization of sperm count and/or ability to procreate at 12- and 15-years following treatment.  Serum LH and FSH were studied in n=11 who also had semen analyses. Sexual maturation was reached from all boys without any need of androgen replacement. | No | Data on gonadal function were available for 20 boys; n=5 were considered prepubescent; no clinical evidence of sexual maturation and <13 years of age.  Both prepubescent and post-pubescent groups of boys were affected by 6 cycles of MOPP, whether pelvic radiation was administered or not. |
| Green, D. M, Hall, B & Zevon, M. A, 1989. Pregnancy outcome after treatment for acute lymphoblastic leukemia during childhood or adolescence. *Cancer,* 64(11), pp.2335–2339. | | | | | |
| Single-centre cohort | Survivors: 44.  Age at diagnosis:  median age of 9.8 years (range, 3.8-15.6 years).  Diagnosis:  Acute Lymphoblastic Leukemia (ALL). | Chemotherapy:  The patients were treated according to various protocols, including ALGB 6307 (n=1), ALGB 6313 (n=1), CALGB 6601 (n=1), CALGB 6801 (n=2), CALGB 7111 (n=2), PPC 429 (n=1), and PPC 498 (n=3). One patient was treated off protocol.  The range of cumulative doses received were methotrexate, 150 to 12,347 mg/m^2^ (median, 4,520 mg/m^2^) (n=12): 6-mercaptopurine, 2,500 to 130,393 mg/m^2^ (median, 98,961 mg/m^2^) (n=10) vincristine, 6 to 76 mg/m^2^ (median, 54 mg/m^2^) (n=12) daunorubicin, 180 to 950 mg/m^2^ (n=2); cyclophosphamide, 1,100 to 17,150 mg/m^2^ (n=2); BCNU, 233 to 270 mg/m^2^ (n=2); and 1-asparaghase, 30,000 to 360,000 U/m^2^ (median, 150,000 U/m^2^) (n=7). | Pregnancy outcomes:  39/44 patients (88%) reported pregnancy outcomes, with 27 pregnancies reported by 8 women and 4 men. The spouses of 4 men, reported 10 pregnancies.  Reporting of one spontaneous abortion; one infant stillborn due to shoulder dystocia; 8 liveborn infants (4 males, 4 females) with median birthweight of 3714 g (range, 3147 to 4536); the birthweight of a single full-term infant was unknown. It is important to also that none of the infants had a birthweight of <2500 g; no congenital anomalies were reported; none has been diagnosed with childhood cancer; currently 1 month to 8 years, 11 months of age (median, 6 years, 1 month). | No |  |
| Jaffe, N., Sullivan, M., Ried, H., Boren, H., Marshall, R., Meistrich, M., Maor, M. and Cunha, M., 1988. Male reproductive function in long-term survivors of childhood cancer. *Medical and Pediatric Oncology*, 16(4), pp.241-247. | | | | | |
| Single-centre cohort | Survivors: 27 males (n=13 were ≤12 years of age).  Age at diagnosis: Prepubertal <12 and Post pubertal group 13-16 years of age.  Diagnosis:  Hodgkin’s Disease and others including a variety of solid tumors, lymphoma, and leukemia. | Chemotherapy was calculated according to the cumulative amount of drug administered.  Treatment:  n=7 received 2-6 cycles of MOPP chemotherapy (nitrogen mustard, oncovin, prednisone and procarbazine); n=5 received COPP (cyclophosphamide, oncovin, prednisone, and procarbazine) or chlorambucil. Among the remaining cohort, n=10 received radiation therapy (n=5 to the inguinal or pelvic nodes) and n=7, an alkylating agent (cyclophosphamide, nitrogen mustard, or chlorambucil); a single leukemia patient with testicular relapse received radiation to the gonads (2,400 rad); n=4 received Adriamycin.  Estimated gonadal scatter radiation dose calculated and ranged from 8-414 rad. | One of 23 patients had a sperm count >100 million; one fathered normal children; n=2 patients had sperm counts >30 million; they were classified as ''probably normal", despite not fathering children. Four had sperm counts between 1 and 10 million and classified as hypo/oligospermic, with their potential for reproduction deemed questionable as they could be sub fertile or infertile.  n=1 patient with a sperm count in the same category was considered to have a normal reproductive capacity because he had fathered children; n=14 were considered sterile on the basis of azoospermia.  n=9 with normal or questionable fertility, had a combined testicular volume ≥30 ml; 4/14 men who were sterile also had a testicular volume >30 ml, and the remaining n=10 a volume <30 ml.  Progressed to puberty normally according to Tanner staging.  Gonadotrophins:  Sterile men testosterone levels were not different from those classified as having normal fertility. Levels appeared to be independent of testicular volume, which means that tubular function when compared to Leydig cell activity was more prone to damage. ↑ LH & FSH levels were associated with sterility and reduced testicular volume; notwithstanding, range of values overlapped with values obtained from men with normal fertility and testicular size. | No | Four patients did not agree to provide semen samples, however implied to had fathered normal healthy children.  Larger scatter doses of radiation were received from sterile patients; n=4 also received alkylating agents and procarbazine; n=2 received alkylating agents.  Patients who had managed to father children indicated no abnormalities in their offspring. |
| P Cramer & J M Andrieu, 1985. Hodgkin's disease in childhood and adolescence: results of chemotherapy-radiotherapy in clinical stages IA-IIB. Journal of Clinical Oncology, 3(11), pp.1495–1502. | | | | | |
| Single-centre cohort | Survivors: 42.  Age at diagnosis:  Median 16 years of age (range, 5-19 years).  Diagnosis: Hodgkin’s Disease (HD). | Treatment:  Clinical stages IA and II_2_A: 3 x MOPP courses (mechlorethamine, Oncovin, procarbazine, and prednisone) and supradiaphragmatic radiotherapy (40 Gy), with no laparotomy. Clinical stages II_3+_A and IIB: either 6 x MOPP (H 72), 3 x MOPP, or 3 x CCNU, vinblastine, procarbazine, and prednisone (CVPP) (H 77) and had a laparotomy followed by supradiaphragmatic irradiation and if positive results were obtained for laparotomy then a lumboaortic field followed. Completion of therapy, resulted in 70/72 patients in complete remission (n=1 failure, n=1 death). | Semen analysis:  *Puberty was normal*, but testicular damage was common after MOPP CT; *azoospermia* was the main outcome in patients with seminal analysis. Most of the subjects studied at the follow-up had ↑ basal serum FSH or an exaggerated FSH response to LH-releasing hormone. | No |  |
| Ahmed, S.R et al., 1983. Primary gonadal damage following treatment of brain tumors in childhood. *The Journal of Pediatrics*, 103(4), pp.562–565. | | | | | |
| Single-centre cohort | Survivors: 10 males.  Age at diagnosis: Range 1.3-14 years.  Age at assessment: Range 4.3-19.3 years.  Diagnosis: Medulloblastoma. | Two groups of children considered that were previously treated for medulloblastoma (surgery and postoperative craniospinal irradiation).  3 doses of vincristine per child given weekly (2.0 mg/M^2^ intravenously) during radiation therapy.  Group 1 (n=9) received adjuvant nitrosourea (BCNU) (100 mg/M^2^) chemotherapy, plus vincristine (2.0 mg/M^2^) (every six weeks for 1-2 years after radiotherapy completion) in 4 and CCNU (50 mg/M^2^) (at 6 weekly intervals for 12-18 months); remaining n=3 received procarbazine 50 mg/M^2^ given on days 8 to 22 of each 6-week cycle in addition to the CCNU. | Group 1: gonadal damage with elevated serum FSH and small testes according to stage of pubertal development.  Minimally ↑ serum prolactin in 1/9 children (564 mU/L), and the serum TSH level was ↑ in 3 children (8, 10, and 19 mU/L).  Group 2 (n=8): All children completed pubertal development normally; had adult sized testes; gonadotrophins were normal. | No | Normal ranges for prepubertal boys (n=15; age range 3-12 years):  serum FSH (0.5-3.1 mU/ml) and LH (0.5-2.2 mU/ml)  Normal ranges for early pubertal boys (n=16; age range 11-14 years; stages P2 and P3): serum FSH (1.3-3.8 mU/ml) and LH (0.5-5.2 mU/ml).  Normal ranges for late pubertal boys (n=10; age range 13-18; stages P4 and P5): serum FSH (2-7 mU/ml) and LH (2.1-7.3 mU/ml). |

**Supplementary Table SIV** Risk of bias as assessed in all the included studies.

+ High risk of bias identified

- Low risk of bias identified

? Unclear.

| **Reference Table** | |
| --- | --- |
| **Bias to be assessed across included studies** | **Reasons Considered** |
| **Confounding** | Variation of pubertal stage; irradiation dose as part of the treatment. |
| **Selection of participants** | Whether chemotherapy is repeated after a relapse. |
| **Classification of interventions** | A prerequisite for an appropriate comparison of interventions is that the interventions are well defined e.g., type, setting, dose, frequency, intensity and/or timing of intervention. |
| **Deviations from intended interventions** | Deviations that do not reflect usual practice and affect the outcome. |
| **Missing data** | Loss to follow-up of certain participants; time at which the blood samples were taken; or cut-off hormonal values not clearly illustrated. |
| **Measurement of Outcomes** | Different follow-up time per man or variability in automated laboratory assays. |
| **Selection of the reported results** | If multiple measurements were made, but only one or a subset is reported. |

| **Medrano et al. (2021)** | | |
| --- | --- | --- |
| **Bias** | **Reason** |  |
| **Confounding** | Pubertal status at diagnosis was not clearly illustrated for each patient individually. However, they do clearly illustrate pre-pubertal patients with the range of ages. | **+** |
| **Selection of participants** | Exclusion criteria involved patients with subsequent neoplasms, to be entirely removed from the cohort. | **+** |
| **Classification of interventions** | Bias in classification of interventions. | **?** |
| **Deviations from intended interventions** | No bias introduced due to deviations from intended interventions. | **-** |
| **Missing data** | Cut-off values of hormones are clearly illustrated. | **?** |
| **Measurement of Outcomes** | Difference of follow-up time per man. | **?** |
| **Selection of the reported results** | Evaluated semen analysis mainly in survivors who were expected to have a high risk of infertility i.e., Hodgkin’s Lymphoma with a higher degree of fertility problems. | **?** |

| **Kruseová et al. (2021)** | | |
| --- | --- | --- |
| **Bias** | **Reason** |  |
| **Confounding** | Pubertal status at diagnosis was not clearly illustrated for each patient individually. However, they do clearly illustrate pre-pubertal patients with the range of ages. | **?** |
| **Selection of participants** | Exclusion criteria involved patients with subsequent neoplasms, to be entirely removed from the cohort. | **-** |
| **Classification of interventions** | Bias in classification of interventions. | **+** |
| **Deviations from intended interventions** | No bias introduced due to deviations from intended interventions. | **-** |
| **Missing data** | Cut-off values of hormones are clearly illustrated. | **-** |
| **Measurement of Outcomes** | Difference of follow-up time per man. | **+** |
| **Selection of the reported results** | Evaluated semen analysis mainly in survivors who were expected to have a high risk of infertility i.e., Hodgkin’s Lymphoma with a higher degree of fertility problems. | **+** |

| **Utriainen et al. (2019)** | | |
| --- | --- | --- |
| **Bias** | **Reason** |  |
| **Confounding** | Pubertal status at diagnosis was not clearly illustrated for the patients. However, one could clearly be led to the assumption that at diagnosis all were pre-pubertal (0.2-3.6). | **-** |
| **Selection of participants** | No relapse mentioned in this study. | **-** |
| **Classification of interventions** | Bias in classification of interventions. | **+** |
| **Deviations from intended interventions** | No bias introduced due to deviations from intended interventions. | **-** |
| **Missing data** | Blood samples for pituitary and gonadal hormone analyses were drawn after an overnight fast between 8.30 and 9.15 a.m. on the examination day. | **-** |
| **Measurement of Outcomes** | Difference of follow-up time per man. | **+** |
| **Selection of the reported results** | Bias in the selection of the reported results. | **?** |

| **Beaud et al. (2019)** | | |
| --- | --- | --- |
| **Bias** | **Reason** |  |
| **Confounding** | CCS group was subdivided according to the age at diagnosis relative to the onset of puberty as defined by the US and European consortium: diagnosed before puberty (ages 4–14). This is a generalisation, as Tanner staging differs between individuals. | **+** |
| **Selection of participants** | No relapse mentioned in this study. | **-** |
| **Classification of interventions** | Bias in classification of interventions. | **+** |
| **Deviations from intended interventions** | Bias introduced due to deviations from intended interventions. | **?** |
| **Missing data** | Bias due to missing data i.e., time at which the blood samples were taken. | **+** |
| **Measurement of Outcomes** | All hormones were assayed using immuno-enzymatic chemiluminescent assays. | **-** |
| **Selection of the reported results** | Bias in the selection of the reported results. | **?** |

| **Green et al. (2014)** | | |
| --- | --- | --- |
| **Bias** | **Reason** |  |
| **Confounding** | Pubertal stage is not commented upon, individuals receiving treatment are subdivided according to their age. | **?** |
| **Selection of participants** | No relapse mentioned in this study. | **-** |
| **Classification of interventions** | No bias in classification of interventions. | **-** |
| **Deviations from intended interventions** | Bias due to deviations from intended interventions. | **?** |
| **Missing data** | No loss to follow-up is described of patients. | **-** |
| **Measurement of Outcomes** | Bias in measurement of outcomes. | **?** |
| **Selection of the reported results** | Bias in the selection of the reported results. | **?** |

| **Shiraishi et al. (2014)** | | |
| --- | --- | --- |
| **Bias** | **Reason** |  |
| **Confounding** | Pubertal stage is not commented upon, just individual age at chemotherapy end is shown. | **+** |
| **Selection of participants** | No relapse mentioned in this study. | **-** |
| **Classification of interventions** | While there is a well-defined intervention, the timing which is crucial is not clearly reported. | **+** |
| **Deviations from intended interventions** | Bias due to deviations from intended interventions. | **?** |
| **Missing data** | Time in the day at which blood samples were taken is unclear. | **+** |
| **Measurement of Outcomes** | No significant differences with duration to infertility treatment i.e., follow-up times per man. | **-** |
| **Selection of the reported results** | Bias in the selection of the reported results. | **?** |

| **Hamre et al. (2012)** | | |
| --- | --- | --- |
| **Bias** | **Reason** |  |
| **Confounding** | Total-body- irradiation as part of the treatment. | **+** |
| **Selection of participants** | Patients who had received gonadotoxic treatment for a second malignancy were not included. | **-** |
| **Classification of interventions** | Treatment interventions are well-defined, the timing which is crucial is not clearly reported. | **+** |
| **Deviations from intended interventions** | Unclear if bias due to deviations from intended interventions. | **?** |
| **Missing data** | Blood samples were taken at 08.00 hours on Day 2, after the patients had fasted. | **-** |
| **Measurement of Outcomes** | Follow-up period is different per man. | **+** |
| **Selection of the reported results** | Unclear whether a subset of results is reported. | **?** |

| **Green et al. (2010)** | | |
| --- | --- | --- |
| **Bias** | **Reason** |  |
| **Confounding** | Infradiaphragmatic irradiation. | **+** |
| **Selection of participants** | Chemotherapy repeated after a relapse. | **+** |
| **Classification of interventions** | Treatment interventions are not well-defined i.e., the timing, frequency of doses, or even the cycles. | **+** |
| **Deviations from intended interventions** | Unclear if bias due to deviations from intended interventions. | **?** |
| **Missing data** | 15% of eligible patients lost to follow-up and 16% declined participation. | **+** |
| **Measurement of Outcomes** | Follow-up period is different per man. | **+** |
| **Selection of the reported results** | Unclear whether a subset of results is reported. | **?** |

| **Zaletel et al. (2010)** | | |
| --- | --- | --- |
| **Bias** | **Reason** |  |
| **Confounding** | Pelvic irradiation in a subset of patients. | **+** |
| **Selection of participants** | Chemotherapy repeated after a relapse. | **+** |
| **Classification of interventions** | Treatment interventions are not well-defined. | **+** |
| **Deviations from intended interventions** | Unclear if bias due to deviations from intended interventions. | **?** |
| **Missing data** | Time in the day at which the blood samples were taken is not illustrated. | **+** |
| **Measurement of Outcomes** | Follow-up time were similar in the group of patients with PH and in the group of patients without endocrinological deficiencies. | **?** |
| **Selection of the reported results** | Unclear whether a subset of results is reported. | **?** |

| **Krawczuk-Rybak et al. (2009)** | | |
| --- | --- | --- |
| **Bias** | **Reason** |  |
| **Confounding** | Childhood patients had been clearly assigned to their subsequent Tanner stages at the time of diagnosis, illustrating their prepubertal status. | **-** |
| **Selection of participants** | All the patients were in first remission. | **-** |
| **Classification of interventions** | Bias in classification of interventions. | **+** |
| **Deviations from intended interventions** | Bias due to deviations from intended interventions. | **?** |
| **Missing data** | Blood tests were done in the morning. | **?** |
| **Measurement of Outcomes** | Variability in automated laboratory assays. | **+** |
| **Selection of the reported results** | Bias in the selection of the reported results. | **?** |

| **Nurmio et al. (2009)** | | |
| --- | --- | --- |
| **Bias** | **Reason** |  |
| **Confounding** | Hematopoietic stem cell transplantation, or cranial irradiation. | **+** |
| **Selection of participants** | Chemotherapy treatments repeated after relapse in some patients still present in the study. | **+** |
| **Classification of interventions** | Treatment interventions are not well-defined. | **+** |
| **Deviations from intended interventions** | Secondary ALL received a high cumulative dose of cyclophosphamide, which is higher than that used in the modern protocols (deviation from intended intervention). | **+** |
| **Missing data** | Time in the day at which the blood samples were taken is unclear. | **+** |
| **Measurement of Outcomes** | No variability in automated laboratory assays for FSH, LH and testosterone. | **-** |
| **Selection of the reported results** | Unclear whether a subset of results is reported. | **?** |

| **Rafsanjani et al. (2007)** | | |
| --- | --- | --- |
| **Bias** | **Reason** |  |
| **Confounding** | Patients who had undergone pelvic irradiation and bone marrow transplantation were excluded. | **-** |
| **Selection of participants** | Chemotherapy treatments repeated after relapse in some patients. | **+** |
| **Classification of interventions** | Treatment interventions are not well-defined i.e., in terms of timings/ frequency. | **+** |
| **Deviations from intended interventions** | Unclear if bias due to deviations from intended interventions. | **?** |
| **Missing data** | Cut-off values of hormones clearly illustrated. | **-** |
| **Measurement of Outcomes** | Unclear whether the years after initial diagnosis and follow-up are significantly different per individual. | **?** |
| **Selection of the reported results** | Unclear whether a subset of results is reported. | **?** |

| **Garolla et al. (2006)** | | |
| --- | --- | --- |
| **Bias** | **Reason** |  |
| **Confounding** | No radiation as part of the regimens. | **-** |
| **Selection of participants** | No relapse and chemotherapy treatment is reported. | **-** |
| **Classification of interventions** | Bias in classification of interventions. | **-** |
| **Deviations from intended interventions** | Bias due to deviations from intended interventions. | **?** |
| **Missing data** | Loss to follow-up of certain participants. | **+** |
| **Measurement of Outcomes** | Variability in automated laboratory assays. | **+** |
| **Selection of the reported results** | Bias in the selection of the reported results. | **-** |

| **Alebouyeh et al. (2005)** | | |
| --- | --- | --- |
| **Bias** | **Reason** |  |
| **Confounding** | Reduced dose radiotherapy to the: upper mantel, spleen and para-aortic lymph nodes. | **-** |
| **Selection of participants** | Chemotherapy repeated after relapse. | **+** |
| **Classification of interventions** | Treatment interventions are not well-defined (in terms of timings or frequency of each drug as part of the regimen). | **+** |
| **Deviations from intended interventions** | Unclear if bias due to deviations from intended interventions. | **?** |
| **Missing data** | Loss to follow-up was not reported. | **-** |
| **Measurement of Outcomes** | 5 and 16-year follow-up studies are the same for all individuals. | **-** |
| **Selection of the reported results** | Unclear whether a subset of results is reported. | **?** |

| **Hobbie et al. (2005)** | | |
| --- | --- | --- |
| **Bias** | **Reason** |  |
| **Confounding** | Patients who had undergone pelvic irradiation and those who had undergone stem cell transplantation were excluded. | **-** |
| **Selection of participants** | No relapse and chemotherapy treatment is reported. | **-** |
| **Classification of interventions** | Bias in classification of interventions. | **+** |
| **Deviations from intended interventions** | Bias due to deviations from intended interventions. | **?** |
| **Missing data** | The time at which blood samples were collected for basal hormone quantification in a day were not illustrated. | **+** |
| **Measurement of Outcomes** | Variable follow-up times per man. | **+** |
| **Selection of the reported results** | Bias in the selection of the reported results. | **?** |

| **Kenney et al. (2001)** | | |
| --- | --- | --- |
| **Bias** | **Reason** |  |
| **Confounding** | Excluded patients who received direct radiotherapy to the pelvis or gonads. | **-** |
| **Selection of participants** | Relapse/recurrence and chemotherapy treatment is given again. | **+** |
| **Classification of interventions** | Bias in classification of interventions. | **+** |
| **Deviations from intended interventions** | Bias due to deviations from intended interventions, unbalanced between the two groups and likely to have affected the outcome. | **+** |
| **Missing data** | The time at which blood samples were collected for basal hormone quantification in a day were not illustrated. Interpretation of the results from the current study is also limited because of the large number of eligible patients who did not participate. | **+** |
| **Measurement of Outcomes** | Established laboratory normal range of values for hormones. | **-** |
| **Selection of the reported results** | Bias in the selection of the reported results. | **?** |

| **Arush et al. (2000)** | | |
| --- | --- | --- |
| **Bias** | **Reason** |  |
| **Confounding** | Nine patients were identified as prepubertal according to their Tanner stage. | **-** |
| **Selection of participants** | No relapse and chemotherapy treatment is reported. | **-** |
| **Classification of interventions** | Bias in classification of interventions. | **+** |
| **Deviations from intended interventions** | Bias due to deviations from intended interventions. | **?** |
| **Missing data** | Loss to follow-up of patients. | **+** |
| **Measurement of Outcomes** | Cut-off points given for normal hormone levels. | **-** |
| **Selection of the reported results** | Bias in the selection of the reported results. | **?** |

| **Heikens et al. (1996)** | | |
| --- | --- | --- |
| **Bias** | **Reason** |  |
| **Confounding** | Two patients were irradiated below the diaphragm (20 Gy on the paraaortal and splenic regions, respectively, and 25 Gy on the inguinal region). | **+** |
| **Selection of participants** | No relapse and chemotherapy treatment is reported. | **-** |
| **Classification of interventions** | Bias in classification of interventions. | **+** |
| **Deviations from intended interventions** | Bias due to deviations from intended interventions. | **?** |
| **Missing data** | The time at which blood samples were collected for basal hormone quantification in a day were not illustrated. | **+** |
| **Measurement of Outcomes** | Variability in automated laboratory assays (different forms of radioimmunoassay for each hormone). | **+** |
| **Selection of the reported results** | Bias in the selection of the reported results. | **?** |

| **Müller et al. (1996)** | | |
| --- | --- | --- |
| **Bias** | **Reason** |  |
| **Confounding** | Gonadal radiotherapy received by some patients. | **+** |
| **Selection of participants** | Chemotherapy treatment is repeated after relapse (cyclophosphamide). | **+** |
| **Classification of interventions** | Treatment interventions are not well-defined i.e., in terms of timings/ frequency. | **+** |
| **Deviations from intended interventions** | Unclear if bias due to deviations from intended interventions. | **?** |
| **Missing data** | Illustrated that the blood samples were obtained 30 and 60 min after injection of GnRH for measurement of stimulated hormone levels for LH and FSH. The basal levels of hormones were measured in serum 20 min before injection, but not mentioning at what time of the day. | **+** |
| **Measurement of Outcomes** | All hormones were measured by radioimmunoassay with commercial kits. | **-** |
| **Selection of the reported results** | A subset of results is reported. No differences between LTS and normal controls of the same sex were found for serum concentrations of testosterone, but the results are not reported. | **+** |

| **Dhabhar et al. (1993)** | | |
| --- | --- | --- |
| **Bias** | **Reason** |  |
| **Confounding** | Patients receiving radiation to the infradiafragmatic region were excluded. | **-** |
| **Selection of participants** | No relapse and chemotherapy treatment is reported. | **-** |
| **Classification of interventions** | Bias in classification of interventions. | **-** |
| **Deviations from intended interventions** | Bias due to deviations from intended interventions. | **?** |
| **Missing data** | But the time at which these blood samples were collected in a day were not illustrated. | **+** |
| **Measurement of Outcomes** | No variability in automated laboratory assays as reproductive hormones said to be quantified with their respective radioimmunoassays. | **-** |
| **Selection of the reported results** | Bias in the selection of the reported results. | **-** |

| **Sy Ortin et al. (1990)** | | |
| --- | --- | --- |
| **Bias** | **Reason** |  |
| **Confounding** | Administration of pelvic irradiation in some patients with no testicular shield at the time of treatment. | **+** |
| **Selection of participants** | Chemotherapy is repeated after a relapse in a prepubertal patient. | **+** |
| **Classification of interventions** | Bias in classification of interventions, as no dose of MOPP administered is reported or the timing of the intervention. | **+** |
| **Deviations from intended interventions** | Bias due to deviations from intended interventions. | **?** |
| **Missing data** | Gonadal function data were not present for most patients treated with HD; loss to follow-up of certain participants. | **+** |
| **Measurement of Outcomes** | Different follow-up time per man. | **+** |
| **Selection of the reported results** | Bias in the selection of the reported results. | **-** |

| **Green et al. (1989)** | | |
| --- | --- | --- |
| **Bias** | **Reason** |  |
| **Confounding** | None of the subjects reporting pregnancy had received craniospinal irradiation. | **-** |
| **Selection of participants** | No relapse mentioned in the study to introduce a bias in the incorporation of participants. | **-** |
| **Classification of interventions** | Treatment interventions are not well-defined i.e., the frequency or timings. | **+** |
| **Deviations from intended interventions** | Unclear if bias due to deviations from intended interventions. | **?** |
| **Missing data** | Bias due to missing data (i.e., loss to follow-up of certain participants). | **?** |
| **Measurement of Outcomes** | Unclear whether the years after initial diagnosis and follow-up are significantly different per individual. | **?** |
| **Selection of the reported results** | Unclear whether a subset of results is reported. | **?** |

| **Jaffe et al. (1988)** | | |
| --- | --- | --- |
| **Bias** | **Reason** |  |
| **Confounding** | Sterility was observed in patients due to testicular radiation alone or in combination with chemotherapy. | **+** |
| **Selection of participants** | Chemotherapy is not repeated post-relapse. | **-** |
| **Classification of interventions** | Bias in classification of interventions. | **+** |
| **Deviations from intended interventions** | Bias introduced due to deviations from intended interventions. | **?** |
| **Missing data** | Loss to follow-up of patients. | **+** |
| **Measurement of Outcomes** | Difference of follow-up time per man. | **?** |
| **Selection of the reported results** | Bias in the selection of the reported results. | **?** |

| **Cramer et al. (1985)** | | |
| --- | --- | --- |
| **Bias** | **Reason** |  |
| **Confounding** | Irradiation as part of the treatment. | **+** |
| **Selection of participants** | Treatment repetition post-relapse. | **+** |
| **Classification of interventions** | Bias in classification of interventions. | **+** |
| **Deviations from intended interventions** | Bias introduced due to deviations from intended interventions. | **?** |
| **Missing data** | Loss to follow-up of patients. | **+** |
| **Measurement of Outcomes** | Difference of follow-up time per man. | **+** |
| **Selection of the reported results** | Bias in the selection of the reported results. | **?** |

| **Anelli et al. (1984)** | | |
| --- | --- | --- |
| **Bias** | **Reason** |  |
| **Confounding** | Patients divided in age groups however might have variation of pubertal stage, as Tanner staging is not performed. | **?** |
| **Selection of participants** | More aggressive chemotherapy repetition after a relapse for patient D, who is termed pubertal but age 12. | **+** |
| **Classification of interventions** | Bias in classification of interventions, although referred that patients have been treated with the same combination of chemotherapeutic agents, for the same period and at the same doses. | **+** |
| **Deviations from intended interventions** | Bias due to deviations from intended interventions. | **?** |
| **Missing data** | Time at which the blood samples were taken. | **+** |
| **Measurement of Outcomes** | No variability in automated laboratory assays; LH and FSH measured with radioimmunoassays. | **-** |
| **Selection of the reported results** | Bias in the selection of the reported results. | **?** |

| **Ahmed et al. (1983)** | | |
| --- | --- | --- |
| **Bias** | **Reason** |  |
| **Confounding** | Craniospinal irradiation as a confounding factor. | **+** |
| **Selection of participants** | No relapse is described in the current study. | **-** |
| **Classification of interventions** | No bias in the classification of interventions. | **-** |
| **Deviations from intended interventions** | Bias due to deviations from intended interventions. Difference in irradiation practises and calculated scatter doses of irradiation to the gonad, where they expected the gonadal dose to be much lower than the maximum quoted in nearly all our children. | **+** |
| **Missing data** | No bias due to missing data, as cut-off values of hormones are clearly illustrated i.e., normal ranges for basal serum FSH and LH for prepubertal boys. | **-** |
| **Measurement of Outcomes** | No bias in the measurement of outcomes, as for quantifying hormones they used radioimmunoassays. | **-** |
| **Selection of the reported results** | Analytic strategies were performed for dealing with analysis of results, i.e., application of methods to generate estimates of the effect of the intervention of irradiation. | **-** |
